# Supplementary material for: Hybrid Models and Biological Model Reduction with PyDSTool
Source: PLoS Comput Biol. 2012 Aug 9;8(8):e1002628. doi: 10.1371/journal.pcbi.1002628 (PMC3415397; doi:10.1371/journal.pcbi.1002628)
Supplement: Text S4 — Complete source code for the PyDSTool package (version 0.88.120504). Includes API documentation and help files linking to web pages. This file is identical to the current public release on Sourceforge.net. (ZIP) [file pcbi.1002628.s004.zip › PyDSTool/html/PyDSTool.Symbolic-pysrc.html]

xml version="1.0" encoding="ascii"?


PyDSTool.Symbolic


| Home | Trees | Indices | Help | PyDSTool |
| --- | --- | --- | --- | --- |

Package PyDSTool ::
Module Symbolic

[hide private]

[frames] | no frames]

# Source Code for Module PyDSTool.Symbolic

```
   1  """Symbolic expression support, and associated utilities. 
   2   
   3  Robert Clewley, October 2005. 
   4   
   5     Includes code for symbolic differentiation by Pearu Peterson 
   6     and Ryan Gutenkunst. 
   7   
   8   
   9   Symbolic Differentiation code: 
  10      Copyright 1999 Pearu Peterson, <pearu@ioc.ee> 
  11      March 11-12, 1999 
  12      Pearu Peterson 
  13   
  14      Modified by Ryan Gutenkunst, <rng7@cornell.edu> 
  15      April 28th, 2005 
  16      General clean-up of code. 
  17      Also changed saving mechanism to explicitly save the results, 
  18      rather than doing it each time a derivative is taken. 
  19   
  20      Modified by Robert Clewley, <rhc28@cornell.edu> 
  21      September 15th, 2005 
  22      As this branch of Peterson's development of symbolic features in Python 
  23        seems to be stopped, I have increased the version number to 0.3 from 0.2. 
  24      Added division by zero check in function dodiv(). 
  25      Added decimal point to integer constants in division to avoid Python 
  26        integer division rules being applied to the expressions. This involves 
  27        an added optional argument to trysimple(), and a new function 
  28        ensuredecimalconst(). This option is conrolled using the global DO_DEC 
  29        switch. 
  30      Added functionality in dofun() for using "pow(x,y)" (with two arguments only) 
  31        in input expressions. 
  32      Also, added DO_POW option to make all output involving powers to use this 
  33        "pow(x,y)" syntax, rather than "x**y". (Set the global DO_POW switch). 
  34      dopower(), domul(), etc. also accept decimals 1.0 and 0.0 to perform 
  35        simplifications if the DO_DEC switch is set. 
  36      Adapted Diff function to accept PyDSTool Var and QuantSpec types, and 
  37        always return those types, and also to accept an array (acts like Jacobian). 
  38        Code is entirely backwards compatible if PyDSTool is not used (except 
  39        Jacobians won't work). 
  40      Renamed original, pure-string Diff function to DiffStr. 
  41      test3() augmented with test for "pow(x,y)" syntax. 
  42      Added test4() for Diff as Jacobian and using Var and QuantSpec objects, 
  43        if available. 
  44      Note that with DO_DEC option off you get "incorrect" results for the derivative 
  45        of f[1] in test4(). 
  46   
  47      25 February 2006 
  48      Moved package into Symbolic.py 
  49      Added scopes to eval statements to prevent argument names clashing with 
  50        names appearing in the eval'd string. 
  51      Made title case symbolic versions of math functions and constants 
  52        from ModelSpec compatible with Diff. 
  53      Improved support for pow(x,y) syntax under differentiation. 
  54      Added support for symbolic vectors. 
  55  """ 
  56   
  57  # ---------------------------------------------------------------------------- 
  58   
  59  from __future__ import division 
  60  import os, sys, types, cPickle 
  61  from Interval import Interval 
  62  from common import * 
  63  from utils import info as utils_info 
  64  from parseUtils import * 
  65  from errors import * 
  66   
  67  #from math import * 
  68  from utils import * 
  69  from numpy import array, Inf, NaN, isfinite, mod, sum, float64, int32 
  70  from numpy import sometrue, alltrue 
  71  # replacements of math functions so that expr2fun etc. produce vectorizable math functions 
  72  from numpy import arccos, arcsin, arctan, arctan2, arccosh, arcsinh, arctanh, ceil, cos, cosh 
  73  from numpy import exp, fabs, floor, fmod, frexp, hypot, ldexp, log, log10, modf, power 
  74  from numpy import sin, sinh, sqrt, tan, tanh 
  75  # for compatibility with numpy 1.0.X 
  76  from math import degrees, radians 
  77  # constants 
  78  from math import pi, e 
  79   
  80  from copy import copy, deepcopy 
  81  import math, random, numpy 
  82   
  83  # alternative names for inverse trig functions, to be supported by expr2fun, symbolic eval, etc. 
  84  asin = arcsin 
  85  acos = arccos 
  86  atan = arctan 
  87  atan2 = arctan2 
  88  acosh = arccosh 
  89  asinh = arcsinh 
  90  atanh = arctanh 
  91  pow = power 
  92   
  93  # --------------------------------------------------------------------------- 
  94  ### Constants 
  95  # determine what "globals" will be for math evaluations in _eval method 
  96  math_dir = dir(math) 
  97  math_globals = dict(zip(math_dir,[getattr(math, m) for m in math_dir])) 
  98  math_globals['Inf'] = Inf 
  99  math_globals['NaN'] = NaN 
 100   
 101  # protected names come from parseUtils.py 
 102  allmathnames = [a for a in protected_mathnames + protected_randomnames + \ 
 103                  ['abs', 'pow', 'min', 'max', 'sum'] if not a.isupper()] 
 104  allmathnames_symbolic = [a.title() for a in allmathnames] 
 105   
 106  # the definitions for the math names are made lower down in this file 
 107  mathNameMap = dict(zip(allmathnames_symbolic, allmathnames)) 
 108  inverseMathNameMap = dict(zip(allmathnames, allmathnames_symbolic)) 
 109   
 110  specTypes = ('RHSfuncSpec', 'ImpFuncSpec', 'ExpFuncSpec') 
 111   
 112   
 113  # -------------------------------------------------------------------------- 
 114  ### Exports 
 115  _functions = ['isMultiDef', 'isMultiRef', 'isMultiDefClash', 'checkbraces', 
 116                'findMultiRefs', 'processMultiRef', 'evalMultiRefToken', 
 117                'expr2fun', 'subs', 'ensureStrArgDict', 'ensureQlist', 
 118  ##              'loadDiffs', 'saveDiffs', 
 119                'Diff', 'DiffStr', 'prepJacobian'] 
 120   
 121  _classes = ['QuantSpec', 'Quantity', 'Var', 'Par', 'Input', 'Fun'] 
 122   
 123  _constants = ['specTypes', 'allmathnames_symbolic', 'allmathnames'] \ 
 124                  + allmathnames_symbolic + ['mathNameMap', 'inverseMathNameMap'] 
 125   
 126  __all__ = _functions + _classes + _constants 
 127   
 128   
 129  # ----------------------------------------------------------------------------- 
 130  # create namemap for title-case to lower case for evaluation 
 131  # to float attempt 
 132  feval_map_const = {'e': repr(e), 'pi': repr(pi)} 
 133  feval_map_symb = {} 
 134  for symb in allmathnames_symbolic: 
 135      feval_map_symb[symb] = symb.lower() 
 136   
 137   
 138  # ----------------------------------------------------------------------------- 
 139  ## Enable mathematical functions to be used like QuantSpecs when building 
 140  ## model specs, by substituting overloaded objects with the same names in 
 141  ## their place. 
 142   
 143  mathlookup = {}.fromkeys(protected_mathnames, 'math.') 
 144  randomlookup = {}.fromkeys(protected_randomnames, 'random.') 
 145  builtinlookup = {'abs': '', 'pow': '', 'max': '', 'min': '', 'sum': ''} 
 146  modlookup = {} 
 147  modlookup.update(mathlookup) 
 148  modlookup.update(randomlookup) 
 149  modlookup.update(builtinlookup) 
 150   
 151  # only rename actual functions (or callable objects) 
 152  funcnames = [n for n in allmathnames if hasattr(eval(modlookup[n]+n), 
 153                                                  "__call__")] 
 154   
 155  # wrapper and overloader class 


156 -class _mathobj(object):


157 -    def __init__(self, name):


158          self.name = name.title() 
 159          if name not in funcnames: 
 160              raise ValueError("This class is intended for mathematical " 
 161                               "functions only")

 162   


163 -    def __call__(self, *argtuple):


164          args = list(argtuple) 
 165          isQuantity = [compareClassAndBases(a,Quantity) for a in args] 
 166          isQuantSpec = [compareClassAndBases(a,QuantSpec) for a in args] 
 167          if len(args) == 0: 
 168              return QuantSpec("__result__", self.name) 
 169          elif sometrue(isQuantity+isQuantSpec): 
 170              if not sometrue(isQuantity): 
 171                  # can only make this evaluate to a number if args is only 
 172                  # made up of numbers and QuantSpecs that are in allmathnames. 
 173                  convert = True   # initial value 
 174                  for i in range(len(args)): 
 175                      if isQuantSpec[i]: 
 176                          if args[i].subjectToken in allmathnames: 
 177                              # convert into original math name 
 178                              args[i] = eval(args[i].subjectToken.lower()) 
 179                          else: 
 180                              # if any one argument or used symbol is a 
 181                              # non-mathname QS then no conversion -- return a QS 
 182                              symbs = args[i].usedSymbols 
 183                              allnonnumbers = [n for n in symbs if isNameToken(n)] 
 184                              if len(allnonnumbers) > 0 \ 
 185                                 and alltrue([n in allmathnames for n in allnonnumbers]): 
 186                                  namemap = dict(zip(allnonnumbers, 
 187                                                 [a.lower() for a in allnonnumbers])) 
 188                                  args[i].mapNames(namemap) 
 189                                  args[i] = eval(args[i]()) 
 190                              else: 
 191                                  convert = False 
 192                                  break 
 193              else: 
 194                  convert = False 
 195              argstr = ", ".join(map(str,args)) 
 196              if convert: 
 197                  try: 
 198                      return apply(eval(self.name.lower()), args) 
 199                  except TypeError: 
 200                      print "Failed to evaluate function %s on args:"%self.name.lower(), argstr 
 201                      raise 
 202              else: 
 203                  return QuantSpec("__result__", self.name + "(" + argstr +")") 
 204          else: 
 205              arglist = [] 
 206              for a in args: 
 207                  if isinstance(a, str): 
 208                      arglist.append(float(a)) 
 209                  else: 
 210                      arglist.append(a) 
 211              try: 
 212                  return float(apply(eval(self.name.lower()), arglist)) 
 213              except NameError: 
 214                  argstr = ", ".join(map(str,args)) 
 215                  return QuantSpec("__result__", self.name + "(" + argstr +")") 
 216              except TypeError: 
 217                  print "Error evaluating %s on args:"%self.name.lower(), args 
 218                  raise

 219   


220 -    def __str__(self):


221          return self.name

 222   


223 -    def __repr__(self):


224          return self.name + " (ModelSpec wrapper)"

 225   
 226   
 227  # now assign all the function names to _mathobj versions 
 228  for f in funcnames: 
 229      if f == 'random': 
 230          # skip this because it's also a module name and it messes around 
 231          # with the namespace too much 
 232          continue 
 233      exec(str(f).title() + " = _mathobj('"+f+"')") 
 234      math_globals[str(f).title()] = eval(str(f).title()) 
 235  # assignment of constants' names continues after QuantSpec is defined 
 236   
 237  # ------------------------------------------------------------------------- 
 238   


239 -def resolveSpecTypeCombos(t1, t2):


240      """Resolve Quantity and QuantSpec types when combined. 
 241       QuantSpec <op> QuantSpec -> specType rules: 
 242       same types -> that type 
 243       ExpFuncSpec o RHSfuncSpec -> RHSfuncSpec 
 244       ImpFuncSpec o RHSfuncSpec -> RHSfuncSpec (should this be an error?) 
 245       ImpFuncSpec o ExpFuncSpec -> ImpFuncSpec""" 
 246      if t1=='RHSfuncSpec' or t2=='RHSfuncSpec': 
 247          return 'RHSfuncSpec' 
 248      elif t1=='ImpFuncSpec' or t2=='ImpFuncSpec': 
 249          return 'ImpFuncSpec' 
 250      else: 
 251          return 'ExpFuncSpec'

 252   
 253   


254 -def getdim(s):


255      """If s is a representation of a vector, return the dimension.""" 
 256      if len(s) > 4 and s[0]=='[' and s[-1]==']': 
 257          return len(splitargs(s[1:-1], ['(','['], [')',']'])) 
 258      else: 
 259          return 0

 260   
 261   


262 -def strIfSeq(x):


263      """Convert sequence of QuantSpecs or strings to a string representation of 
 264      that sequence, otherwise return the argument untouched.""" 
 265      if isinstance(x, _seq_types): 
 266          if len(x) > 0: 
 267              xstrlist = map(str,x) 
 268              return '['+",".join(xstrlist)+']' 
 269          else: 
 270              return x 
 271      else: 
 272          return x

 273   
 274   


275 -def ensureQlist(thelist):


276      o = [] 
 277      for s in thelist: 
 278          if isinstance(s, str): 
 279              o.append(QuantSpec('__dummy__', s)) 
 280          elif isinstance(s, QuantSpec) or isinstance(s, Quantity): 
 281              o.append(s) 
 282          else: 
 283              raise TypeError("Invalid type for symbolic quantities list") 
 284      return o

 285   
 286   


287 -def ensureStrArgDict(d, renderForCode=True):


288      """Optional renderForCode argument converts any uppercase built-in 
 289      symbolic function names to lowercase numeric function names 
 290      (default True)""" 
 291      o = {} 
 292      try: 
 293          # d is a Point or dictionary 
 294          for k, v in d.iteritems(): 
 295              if isinstance(v, tuple): 
 296                  if alltrue([isinstance(x, str) for x in v[0]]): 
 297                      o[str(k)] = (v[0], str(v[1])) 
 298                  else: 
 299                      o[str(k)] = ([str(x) for x in v[0]], str(v[1])) 
 300              else: 
 301                  o[str(k)] = str(v) 
 302      except AttributeError: 
 303          # d is not a Point or Dictionary 
 304          if isinstance(d, list): 
 305              for q in d: 
 306                  o.update(ensureStrArgDict(q)) 
 307          else: 
 308              try: 
 309                  if hasattr(d, 'signature'): 
 310                      # q is a Fun Quantity 
 311                      if renderForCode: 
 312                          o[d.name] = (d.signature, str(d.spec.renderForCode())) 
 313                      else: 
 314                          o[d.name] = (d.signature, d.spec.specStr) 
 315                  elif hasattr(d, 'name'): 
 316                      # d is any other type of Quantity 
 317                      if renderForCode: 
 318                          o[d.name] = str(d.spec.renderForCode()) 
 319                      else: 
 320                          o[d.name] = d.spec.specStr 
 321                  elif hasattr(d, 'subjectToken'): 
 322                      # d is a QuantSpec 
 323                      if renderForCode: 
 324                          o[d.subjectToken] = str(d.renderForCode()) 
 325                      else: 
 326                          o[d.subjectToken] = d.specStr 
 327                  else: 
 328                      raise TypeError("Invalid type %s"%str(type(d))) 
 329              except TypeError, err: 
 330                  print "Argument was:\n", d 
 331                  raise TypeError("Invalid argument type: %s "%str(err) + \ 
 332                     "or argument's values cannot be converted to key:value" + \ 
 333                                  "strings") 
 334      return o

 335   
 336   


337 -def _eval(q, math_globals, local_free, eval_at_runtime):


338      if isinstance(q, str): 
 339          qstr = q 
 340      else: 
 341          qstr = str(q) 
 342      try: 
 343          val = eval(qstr, math_globals, local_free) 
 344      except NameError, err: 
 345          raise ValueError("Invalid input.\nProblem was: %s"%str(err)) 
 346      except TypeError, err: 
 347          # trap getitem or call error for string version of object 
 348          # try without runtime components, if any 
 349          if eval_at_runtime != []: 
 350              try: 
 351                  val = eval(qstr, math_globals, 
 352                             filteredDict(local_free, eval_at_runtime, neg=True)) 
 353              except (NameError, TypeError): 
 354                  val = q 
 355          else: 
 356              val = q 
 357      except KeyboardInterrupt: 
 358          raise 
 359      except: 
 360          print "Internal error evaluating function spec" 
 361          info(local_free, " ** Local free dict was") 
 362          info(math_globals, " ** Math globals dict was") 
 363          raise 
 364      return val

 365   
 366   


367 -def _propagate_str_eval(s):


368      if isinstance(s, list): 
 369          return "["+",".join([_propagate_str_eval(s_el) for s_el in s])+"]" 
 370      elif isinstance(s, tuple): 
 371          return "("+",".join([_propagate_str_eval(s_el) for s_el in s])+")" 
 372      elif isinstance(s, str): 
 373          return s 
 374      else: 
 375          raise TypeError("Don't know how to evaluate to string: %s"%(str(s)))

 376   
 377   


378 -def _join_sublist(qspec, math_globals, local_free, eval_at_runtime):


379      if qspec.isvector(): 
 380          sl = qspec.fromvector() 
 381          # sl is guaranteed to be QuantSpecs 
 382          return "["+",".join([str(_eval(q, math_globals, local_free, 
 383                          eval_at_runtime)) for q in sl])+"]" 
 384      else: 
 385          return str(_eval(qspec, math_globals, local_free, eval_at_runtime))

 386   
 387   


388 -def expr2fun(qexpr, ensure_args=None, **values):


389      """qexpr is a string, Quantity, or QuantSpec object. 
 390      values is a dictionary binding free names to numerical values 
 391      or other objects defined at runtime that are in local scope. 
 392   
 393      If values contains pairs (fnsig, fndef) from a FuncSpec function 
 394      definition then the function will be incorporated into the result. 
 395      The function definition can contain math names that are still 
 396      in non-code-rendered (lowercase) form (i.e. "Exp" rather than "exp"). 
 397   
 398      If any names use the hierarchical naming convention (using the '.' 
 399      separator) then those names will be converted to use the '_' separator 
 400      instead. For end-use convenience, the necessary name map for arguments 
 401      is stored in the '_namemap' attribute, which will otherwise be the ID map. 
 402      Calling with keyed objects that use hierarchical names (especially Point 
 403      objects) can then be done using the return object's alt_call method, 
 404      which takes any keyed_object that will be filtered for the function 
 405      arguments and name-mapped appropriately before evaluation. 
 406   
 407      You can force the function to accept the named arguments in the 
 408      ensure_args list of strings, e.g. when creating a Jacobian 
 409      that is expected to always take 't' as an argument even if 
 410      the system is autonomous. 
 411   
 412      The arguments to the returned callable function object are 
 413      given by its attribute '_args', and its definition string by 
 414      '_call_spec'. 
 415      """ 
 416      # put in local namespace for sake of final class method's 
 417      # alt_call use of filteredDict 
 418      from common import filteredDict 
 419      # convert values into a dictionary of strings where possible 
 420      valDict = {} 
 421      # functions that will end up as methods in the wrapper 
 422      embed_funcs = {} 
 423      local_free = {} 
 424      local_funcs = {}  # temp Fun for eval'ing strings 
 425      eval_at_runtime = [] 
 426      free = [] 
 427      h_map = symbolMapClass() 
 428      for k, v in values.iteritems(): 
 429          if isinstance(v, str): 
 430              vs = v 
 431          elif isinstance(v, _num_types): 
 432              # retain most accuracy in float 
 433              vs = repr(v) 
 434          elif isinstance(v, tuple): 
 435              # ensure it's a valid function definition 
 436              try: 
 437                  fnsig, fndef = v 
 438              except ValueError: 
 439                  raise ValueError("Only pass pairs in 'values' argument that are function definitions") 
 440              try: 
 441                  vs = Fun(fndef, fnsig, k) 
 442              except: 
 443                  raise ValueError("Only pass pairs in 'values' argument that are valid function definitions") 
 444              local_funcs[k] = Var(k)  # placeholder 
 445              eval_at_runtime.append(k) 
 446              for vfree in vs.freeSymbols: 
 447                  if vfree not in values and vfree not in math_globals: 
 448                      raise ValueError("Free name %s inside function %s must be bound" % (vfree, k)) 
 449              embed_funcs[k] = (fnsig, fndef) 
 450          else: 
 451              vs = str(v) 
 452          if isNumericToken(vs): 
 453              if '.' in k: 
 454                  kFS = k.replace('.','_') 
 455                  h_map.update({k: kFS}) 
 456              else: 
 457                  kFS = k 
 458              valDict[kFS] = vs 
 459          elif not isinstance(vs, Fun): 
 460              if '.' in k: 
 461                  kFS = k.replace('.','_') 
 462                  h_map.update({k: kFS}) 
 463              else: 
 464                  kFS = k 
 465              local_free[kFS] = v 
 466              eval_at_runtime.append(kFS) 
 467      # ensure QuantSpec 
 468      if isinstance(qexpr, Quantity): 
 469          qexpr = qexpr.spec 
 470      # now make a Var from the QuantSpec 
 471      try: 
 472          qvar = Var(qexpr, 'q') 
 473      except TypeError, e: 
 474          raise TypeError("Invalid qexpr argument type: " + str(e)) 
 475      fspec = qvar.eval(**valDict) 
 476      for fname, (fnsig, fndef) in embed_funcs.iteritems(): 
 477          qtemp = QuantSpec('q', fndef) 
 478          for emb_fname in embed_funcs.keys(): 
 479              # check for embedded function names in these definitions that need 
 480              # prefix 'self.' for later function creation 
 481              if emb_fname in qtemp: 
 482                  # replace all occurrences 
 483                  qtemp.mapNames({emb_fname: 'self.'+emb_fname}) 
 484          new_fndef = str(qtemp.eval(**filteredDict(valDict, fnsig, neg=True)).renderForCode()) 
 485          embed_funcs[fname] = (fnsig, new_fndef) 
 486      free.extend(remain(fspec.freeSymbols, 
 487                    math_globals.keys()+local_free.keys()+embed_funcs.keys())) 
 488      # hack to exclude object references which will be taken care of at runtime 
 489      # or string literals that aren't supposed to be free symbols 
 490      free = [sym for sym in free if not \ 
 491              ("'%s'"%sym in qexpr or '"%s"'%sym in qexpr)] 
 492      mapnames = local_free.keys() + embed_funcs.keys() 
 493      for k in local_free.keys(): 
 494          for sym in free: 
 495              if '.' in sym and sym[:sym.index('.')] == k: 
 496                  mapnames.append(sym) 
 497                  free.remove(sym) 
 498      # eval simplifies the arithmetic where possible, and defining 
 499      # local QuantSpecs to correspond to the free names allows eval to return 
 500      # a new QuantSpec. 
 501      # 
 502      # first, get rid of hierarchical names before running eval, which will think 
 503      # they are objects! 
 504      free_h = [symb.replace('.', '_') for symb in free] 
 505      free_h_nonFS = [symb.replace('_', '.') for symb in free] 
 506      h_map.update(dict(zip(free_h_nonFS, free_h))) 
 507      free = free_h 
 508      defs = filteredDict(local_free, [k for k, v in local_free.iteritems() if v is not None]) 
 509      local_free.update(dict(zip(free, [QuantSpec(symb) for symb in free]))) 
 510      scope = filteredDict(local_free, [k for k, v in local_free.iteritems() if v is not None]) 
 511      scope.update(local_funcs) 
 512      fspec.mapNames(h_map) 
 513      eval_globals = math_globals.copy() 
 514      eval_globals['max'] = QuantSpec('max', '__temp_max__') 
 515      eval_globals['min'] = QuantSpec('min', '__temp_min__') 
 516      eval_globals['sum'] = QuantSpec('sum', '__temp_sum__') 
 517      if fspec.isvector(): 
 518          # e.g. for a Jacobian or other matrix-valued function 
 519          # expect a list of lists of Quantities (i.e., rank 2 only) 
 520          temp_str = "[" + ",".join([_join_sublist(q, eval_globals, 
 521                            scope, eval_at_runtime) \ 
 522                                     for q in fspec.fromvector()]) + "]" 
 523          fspec_eval = QuantSpec('__temp__', temp_str).renderForCode() 
 524          if eval_at_runtime != []: 
 525              fspec_eval.mapNames(dict(zip(mapnames, 
 526                                      ['self.'+k for k in mapnames]))) 
 527          fspec_str = str(fspec_eval) 
 528      else: 
 529          fspec_eval = _eval(fspec, eval_globals, 
 530                             scope, eval_at_runtime) 
 531      def append_call_parens(k): 
 532          """For defined references to future fn_wrapper attributes that need 
 533          calling to evaluate, append the call parentheses for the text that 
 534          will be embedded in the fn_wrapper definition.""" 
 535          try: 
 536              if defs[k]._args == []: 
 537                  return '()' 
 538              else: 
 539                  return '' 
 540          except (AttributeError, KeyError): 
 541              return ''

 542      if isinstance(fspec_eval, _num_types): 
 543          if eval_at_runtime != []: 
 544              fspec.mapNames(dict(zip(mapnames, 
 545                          ['self.'+k+append_call_parens(k) for k in mapnames]))) 
 546              fspec_str = str(fspec) 
 547          else: 
 548              fspec_str = repr(fspec_eval) 
 549      elif not fspec.isvector(): 
 550          # already found fspec_str using eval_at_runtime for isvector case 
 551          if eval_at_runtime != []: 
 552              fspec.mapNames(dict(zip(mapnames, 
 553                          ['self.'+k+append_call_parens(k) for k in mapnames]))) 
 554              fspec_str = str(fspec) 
 555          else: 
 556              fspec_str = str(fspec_eval.renderForCode()) 
 557      arglist = copy(free) 
 558      if ensure_args is not None: 
 559          arglist.extend(remain(ensure_args, free)) 
 560      if len(arglist) > 0: 
 561          arglist_str = ', ' + ", ".join(arglist) 
 562      else: 
 563          arglist_str = '' 
 564          # only include if the 'function' has no arguments and can 
 565          # be treated as a dynamic-valued variable 
 566      # now that exec has to be guarded with locals(), etc must add these explicit 
 567      # imports as per header of Symbolic.py 
 568      my_locals = locals() 
 569      my_locals.update(math_globals) 
 570      # !!! PERFORM MAGIC 
 571      def_str = """ 
 572  class fn_wrapper(object): 
 573      def __call__(self""" + arglist_str + """): 
 574          return """ + fspec_str + """ 
 575   
 576      def alt_call(self, keyed_arg): 
 577          return self.__call__(**filteredDict(self._namemap(keyed_arg), self._args)) 
 578  """ 
 579      if len(embed_funcs) > 0: 
 580          for fname, (fnsig, fndef) in embed_funcs.iteritems(): 
 581              def_str += "\n    def " + fname + "(self" 
 582              if len(fnsig) > 0: 
 583                  def_str += ", " + ", ".join(fnsig) 
 584              def_str += "):\n        return " + fndef 
 585      try: 
 586          exec def_str in locals(), globals() 
 587      except: 
 588          print "Problem defining function:" 
 589          raise 
 590      evalfunc = fn_wrapper() 
 591      evalfunc.__dict__.update(defs) 
 592      evalfunc._args = arglist 
 593      evalfunc._call_spec = fspec_str 
 594      evalfunc._namemap = h_map 
 595      return evalfunc 
 596   
 597   
 598   


599 -def subs(qexpr, *bindings):


600      """Substitute pars or other name bindings (as a dictionary) 
 601      into a Quantity, QuantSpec, or string containing a symbolic expression.""" 
 602      valDict = {} 
 603      qstype = qexpr.specType 
 604      isQuant = compareBaseClass(qexpr, Quantity) 
 605      if isQuant: 
 606          qtemp = qexpr() 
 607          varname = qexpr.name 
 608      else: 
 609          qtemp = copy(qexpr) 
 610          varname = "" 
 611      for b in bindings: 
 612          if isinstance(b, list): 
 613              # in case called from a function when the bindings 
 614              # cannot be unravelled in the call arguments 
 615              for bentry in b: 
 616                  qtemp = subs(qtemp, bentry) 
 617          elif isinstance(b, Par): 
 618              valDict[str(b)] = str(b.eval()) 
 619          elif isinstance(b, dict): 
 620              for k, v in b.iteritems(): 
 621                  if k in valDict: 
 622                      raise ValueError("Value for %s already bound"%k) 
 623                  valDict[k] = str(v) 
 624          else: 
 625              raise TypeError("Invalid binding type '%s' for substitution"%type(b)) 
 626      tempvar = Var(qtemp, '__result__') 
 627      tempresult = tempvar.eval(**valDict) 
 628      if isQuant: 
 629          result = Var(tempresult, varname) 
 630      else: 
 631          result = tempresult 
 632      result.specType = qstype 
 633      return result

 634   
 635   


636 -def _parse_func_deriv(fname):


637      """Internal utility to test whether a function name is generated 
 638      from a symbolic differentiation call. 
 639      """ 
 640      if '_' in fname: 
 641          # find position from end (in case more than one) 
 642          neg_ix = fname[::-1].index('_') 
 643          # expect integer after _ 
 644          orig_name = fname[:-neg_ix-1] 
 645          int_part = fname[-neg_ix:] 
 646          if len(orig_name) > 0 and len(int_part) > 0 and \ 
 647             isIntegerToken(int_part): 
 648              return (orig_name, int(int_part)) 
 649          else: 
 650              return (fname, None) 
 651      else: 
 652          return (fname, None)

 653   


654 -def _generate_subderivatives(symbols, fnspecs):


655      """Internal utility for prepJacobian""" 
 656      add_fnspecs = {} 
 657      new_free = [] 
 658      math_globals_keys = math_globals.keys() 
 659      for symb in symbols: 
 660          # derivatives of an unknown function f(x,y,z) will be marked 
 661          # f_0, f_1, or f_2 depending on which variable was selected 
 662          orig_name, pos = _parse_func_deriv(symb) 
 663          if pos is not None: 
 664              # then it was a legitimate derivative of function 
 665              try: 
 666                  fnsig, fndef = fnspecs[orig_name] 
 667              except (KeyError, TypeError): 
 668                  raise KeyError("Function %s not present in fnspecs" % orig_name) 
 669              try: 
 670                  var = fnsig[pos] 
 671              except KeyError: 
 672                  # position was not legitimate for this function 
 673                  raise ValueError("Invalid position in signature of function %s" % orig_name) 
 674              Df = Diff(fndef, var) 
 675              add_fnspecs[symb] = (fnsig, str(Df)) 
 676              new_free.extend(remain(Df.freeSymbols, new_free+math_globals_keys)) 
 677      # if any auxiliary functions showed up as free names, add them to add_fnspecs 
 678      for f in new_free: 
 679          if f in fnspecs and f not in add_fnspecs: 
 680              add_fnspecs[f] = fnspecs[f] 
 681      return new_free, add_fnspecs

 682   
 683   


684 -def prepJacobian(varspecs, coords, fnspecs=None, max_iter_depth=20):


685      """Returns a symbolic Jacobian and updated function specs to support 
 686      its definition from variable specifications. Only makes the Jacobian 
 687      with respect to the named coordinates, which will be sorted into 
 688      alphabetical order. 
 689   
 690      """ 
 691      need_specs = filteredDict(varspecs, coords) 
 692      coords.sort() 
 693      jac = Diff(sortedDictValues(need_specs), coords) 
 694      free = jac.freeSymbols 
 695      if fnspecs is None: 
 696          new_fnspecs = {} 
 697      else: 
 698          new_fnspecs = copy(fnspecs) 
 699      # iterate until all inter-function inter-dependencies resolved 
 700      n = 0 
 701      while n < max_iter_depth: 
 702          free, add_fnspecs = _generate_subderivatives(free, new_fnspecs) 
 703          if add_fnspecs == {}: 
 704              break 
 705          else: 
 706              new_fnspecs.update(add_fnspecs) 
 707              n += 1 
 708      return jac, new_fnspecs

 709   
 710   
 711   


712 -def checkbraces(s):


713      """Internal utility to verify that braces match.""" 
 714      if s.count('(') != s.count(')') or \ 
 715         s.count('[') != s.count(']'): 
 716          raise SyntaxError("Mismatch of braces in expression %s" % s)

 717   
 718   
 719  # Local version of who function because it's defined in PyDSTool.__init__ 
 720  # which cannot be imported here (circular import) 
 721  # This function only finds Quantity or QuantSpec objects 


722 -def whoQ(objdict=None, deepSearch=False, frameback=3):


723      objdict_out = {} 
 724      if objdict is None: 
 725          # look a default of 3 stack frames further back to access user's frame 
 726          assert int(frameback)==frameback, "frameback argument must be a positive integer" 
 727          assert frameback > 0, "frameback argument must be a positive integer" 
 728          frame = sys._getframe() 
 729          for i in range(frameback): 
 730              frame = frame.f_back 
 731          objdict = frame.f_globals 
 732      typelist = [Quantity, QuantSpec] 
 733      for objname, obj in objdict.iteritems(): 
 734          if type(obj) not in [type, types.ClassType, types.ModuleType]: 
 735              if compareClassAndBases(obj, typelist): 
 736                  objdict_out[objname] = obj 
 737              elif deepSearch: 
 738                  if isinstance(obj, list) or isinstance(obj, tuple): 
 739                      if sometrue([compareClassAndBases(x, typelist) \ 
 740                                   for x in obj]): 
 741                          objdict_out[objname] = obj 
 742                  elif isinstance(obj, dict): 
 743                      if sometrue([compareClassAndBases(x, typelist) \ 
 744                                   for x in obj.values()]): 
 745                          objdict_out[objname] = obj 
 746      return objdict_out   # original 'returnlevel=2'

 747   
 748  # ---------------------------------------------------------------------- 
 749   
 750  _localfuncnames = protected_macronames + builtin_auxnames 
 751  _avoid_math_symbols = ['e', 'pi'] + allmathnames_symbolic 
 752   


753 -class QuantSpec(object):


754      """Specification for a symbolic numerical quantity.""" 
 755   


756 -    def __init__(self, subjectToken, specStr="", specType=None, 
 757                   includeProtected=True, treatMultiRefs=True, 
 758                   preserveSpace=False, ignoreSpecial=None):


759          if isNameToken(subjectToken) or (isMultiRef(subjectToken) \ 
 760                     and treatMultiRefs and alltrue([char not in subjectToken \ 
 761                                         for char in ['+','-','/','*','(',')']])): 
 762              token = subjectToken.strip() 
 763  ##            if token in ('for','sum'): 
 764  ##                raise ValueError("Cannot use reserved macro name '%s' as a "%token +\ 
 765  ##                                 "QuantSpec's name") 
 766  ##            else: 
 767              self.subjectToken = token 
 768          else: 
 769              print "Found:",subjectToken 
 770              raise ValueError("Invalid subject token") 
 771          if specStr != "" and specStr[0] == '+': 
 772              if len(specStr) > 1: 
 773                  specStr = specStr[1:] 
 774              else: 
 775                  raise ValueError("Invalid specification string") 
 776          if specType is None: 
 777              self.specType = 'ExpFuncSpec' 
 778          elif specType in specTypes: 
 779              self.specType = specType 
 780          else: 
 781              raise ValueError("Invalid specType provided: %s"%specType) 
 782          ignoreTokens = ['[',']'] 
 783          if ignoreSpecial is not None: 
 784              ignoreTokens.extend(remain(ignoreSpecial,ignoreTokens)) 
 785          self.parser = parserObject(specStr, includeProtected, 
 786                                     treatMultiRefs=treatMultiRefs, 
 787                                     ignoreTokens=ignoreTokens, 
 788                                     preserveSpace=preserveSpace) 
 789          self.specStr = "".join(self.parser.tokenized) 
 790          checkbraces(self.specStr) 
 791          # transfer these up to this level 
 792          self.usedSymbols = self.parser.usedSymbols 
 793          self.freeSymbols = self.parser.freeSymbols 
 794          try: 
 795              # assume max of rank 2 
 796              self.dim = getdim(self.specStr)  # for vector 
 797          except IndexError: 
 798              # spec not long enough therefore self cannot be a vector 
 799              self.dim = 0 
 800          self._check_self_ref()

 801   


802 -    def _check_self_ref(self):


803          if self.specType == 'ExpFuncSpec': 
 804              if self.subjectToken in self.parser.usedSymbols: 
 805                  locs = self.parser.find(self.subjectToken) 
 806                  for loc in locs: 
 807                      if loc <= 1 or self.parser.tokenized[loc-2:loc] != ['initcond', '(']: 
 808                          raise ValueError("Cannot define the symbol "+self.subjectToken+" in" 
 809                                   " terms of itself with spec type ExpFuncSpec")

 810   
 811   


812 -    def redefine(self, specStr):


813          p = self.parser 
 814          self.parser = parserObject(specStr, p.includeProtected, 
 815                           p.treatMultiRefs, p.ignoreTokens, p.preserveSpace) 
 816          self.specStr = "".join(self.parser.tokenized) 
 817          checkbraces(self.specStr) 
 818          self.usedSymbols = self.parser.usedSymbols 
 819          self.freeSymbols = self.parser.freeSymbols 
 820          try: 
 821              # assume max of rank 2 
 822              self.dim = getdim(self.specStr)  # for vector 
 823          except IndexError: 
 824              # spec not long enough therefore self cannot be a vector 
 825              self.dim = 0

 826   
 827   


828 -    def isspecdefined(self):


829          return self.specStr.strip() != ""

 830   
 831   


832 -    def isDefined(self, verbose=False):


833          return self.specStr.strip() != ""

 834   
 835   


836 -    def __call__(self, *args):


837          # args is a trick to avoid an exception when Python eval() function 
 838          # is called on an expression involving a function, and the function 
 839          # name has only been defined as a QuantSpec: then this is where eval 
 840          # will be calling, with whatever arguments appeared in the expression! 
 841          # In that case just return the arguments as a string in a string form 
 842          # of the function call (like the identity function) 
 843          if self.isDefined(): 
 844              # get QuantSpec's definition, if it exists 
 845              base_str = self.specStr 
 846          else: 
 847              # otherwise, it may be an empty QuantSpec, like the way 
 848              # pi is defined 
 849              base_str = self.subjectToken 
 850          if args == (): 
 851              return base_str 
 852          else: 
 853              # need to return a QuantSpec type if being called with arguments 
 854              # and propagate str() calls to all sub-elements of any sequence types 
 855              return QuantSpec("__result__", base_str + "(" + \ 
 856                         _propagate_str_eval(args) + ")", self.specType)

 857   
 858   


859 -    def isCompound(self, ops = ['+','-','*','/']):


860          return self.parser.isCompound(ops)

 861   
 862   


863 -    def __contains__(self, tok):


864          return tok in self.parser.tokenized

 865   


866 -    def __getitem__(self, ix):


867          return self.parser.tokenized[ix]

 868   


869 -    def __setitem__(self, ix, s):


870          raise NotImplementedError

 871   


872 -    def __delitem__(self, ix):


873          raise NotImplementedError

 874   
 875      # -------------- method group for spec string assembly 


876 -    def __combine(self, other, opstr, reverseorder=0):


877          # includes dealing with 0 + s -> s, s*0 -> 0, 1 * s -> s, etc. 
 878          # doesn't tackle issues with 0 appearing in a division 
 879          # QuantSpec <op> QuantSpec -> specType rules used by 
 880          # resolveSpecTypeCombos() function: 
 881          #   same types -> that type 
 882          #   ExpFuncSpec o RHSfuncSpec -> RHSfuncSpec 
 883          #   ImpFuncSpec o RHSfuncSpec -> RHSfuncSpec (should this be an error?) 
 884          #   ImpFuncSpec o ExpFuncSpec -> ImpFuncSpec 
 885          specType = self.specType   # default initial value 
 886          self_e = None 
 887          try: 
 888              self_e = eval(self.specStr, {}, {}) 
 889              check_self_e = True 
 890          except: 
 891              check_self_e = False 
 892          include_self = True 
 893          if check_self_e and (isinstance(self_e, _num_types)): 
 894              if self_e == 0: 
 895                  # we don't try to deal with divisions involving 0 here! 
 896                  if opstr == '*': 
 897                      if hasattr(other, 'specType'): 
 898                          specType = resolveSpecTypeCombos(self.specType, 
 899                                                           other.specType) 
 900                      else: 
 901                          specType = self.specType 
 902                      return QuantSpec("__result__", "0", specType) 
 903                  elif opstr in ['+','-']: 
 904                      include_self = False 
 905              elif self_e == 1 and opstr == '*': 
 906                  include_self = False 
 907          if self.isDefined(): 
 908              if reverseorder: 
 909                  if self.specStr[0] == '-' and opstr == '-': 
 910                      opstr = '+' 
 911                      self_specStr_temp = self.specStr[1:] 
 912                  else: 
 913                      self_specStr_temp = self.specStr 
 914              else: 
 915                  self_specStr_temp = self.specStr 
 916          else: 
 917              self_specStr_temp = self.subjectToken 
 918          if isinstance(other, Quantity): 
 919              otherstr = other.name 
 920              otherbraces = 0 
 921              specType = resolveSpecTypeCombos(self.specType, other.specType) 
 922          elif isinstance(other, QuantSpec): 
 923              specType = resolveSpecTypeCombos(self.specType, other.specType) 
 924              e = None 
 925              # get QuantSpec's definition, if it exists 
 926              otherstr = other() 
 927              try: 
 928                  e = eval(otherstr, {}, {}) 
 929                  check_e = True 
 930              except: 
 931                  check_e = False 
 932              if check_e and isinstance(e, _num_types): 
 933                  if e == 0: 
 934                      # we don't try to deal with divisions involving 0 here! 
 935                      if opstr == '*': 
 936                          return QuantSpec("__result__", "0", specType) 
 937                      elif opstr in ['+','-']: 
 938                          return QuantSpec("__result__", self_specStr_temp, 
 939                                           specType) 
 940                      elif opstr == '/' and reverseorder: 
 941                          return QuantSpec("__result__", "0", self.specType) 
 942                  elif e == 1 and (opstr == '*' or \ 
 943                                   (opstr == '/' and not reverseorder)): 
 944                      return QuantSpec("__result__", self_specStr_temp, specType) 
 945                  elif e == -1 and (opstr == '*' or \ 
 946                                   (opstr == '/' and not reverseorder)): 
 947                      return QuantSpec("__result__", '-'+self_specStr_temp, 
 948                                       specType) 
 949              if otherstr[0] == '-' and opstr == '-' and not reverseorder: 
 950                  opstr = '+' 
 951                  otherstr = str(otherstr[1:]) 
 952              otherbraces = int((opstr == '*' or opstr == '-') and other.isCompound(['+','-']) \ 
 953                             or opstr == '/' and other.isCompound()) 
 954          elif isinstance(other, str): 
 955              e = None 
 956              try: 
 957                  e = eval(other, {}, {}) 
 958                  check_e = True 
 959              except: 
 960                  check_e = False 
 961              if check_e and isinstance(e, _num_types): 
 962                  if e == 0: 
 963                      # we don't try to deal with divisions involving 0 here! 
 964                      if opstr == '*': 
 965                          return QuantSpec("__result__", "0", self.specType) 
 966                      elif opstr in ['+','-']: 
 967                          return QuantSpec("__result__", self_specStr_temp, 
 968                                           self.specType) 
 969                      elif opstr == '/' and reverseorder: 
 970                          return QuantSpec("__result__", "0", self.specType) 
 971                  elif e == 1 and (opstr == '*' or \ 
 972                                   (opstr == '/' and not reverseorder)): 
 973                      return QuantSpec("__result__", self_specStr_temp, 
 974                                       self.specType) 
 975                  elif e == -1 and (opstr == '*' or \ 
 976                                   (opstr == '/' and not reverseorder)): 
 977                      return QuantSpec("__result__", '-'+self_specStr_temp, 
 978                                       self.specType) 
 979              if other[0] == '-' and opstr == '-' and not reverseorder: 
 980                  opstr = '+' 
 981                  otherstr = str(other[1:]).strip() 
 982              else: 
 983                  otherstr = str(other).strip() 
 984              temp = QuantSpec("__result__", otherstr, self.specType) 
 985              otherbraces = int((opstr == '*' or opstr == '-') and temp.isCompound(['+','-']) \ 
 986                             or opstr == '/' and temp.isCompound()) 
 987          elif isinstance(other, _num_types): 
 988              if other == 0: 
 989                  # we don't try to deal with divisions involving 0 here! 
 990                  if opstr == '*': 
 991                      return QuantSpec("__result__", "0", self.specType) 
 992                  elif opstr in ['+','-']: 
 993                      return QuantSpec("__result__", self_specStr_temp, 
 994                                       self.specType) 
 995                  elif opstr == '/' and reverseorder: 
 996                      return QuantSpec("__result__", "0", self.specType) 
 997              elif other == 1 and (opstr == '*' or \ 
 998                                   (opstr == '/' and not reverseorder)): 
 999                  return QuantSpec("__result__", self_specStr_temp, 
1000                                   self.specType) 
1001              elif other == -1 and (opstr == '*' or \ 
1002                                   (opstr == '/' and not reverseorder)): 
1003                  return QuantSpec("__result__", '-'+self_specStr_temp, 
1004                                   self.specType) 
1005              if other < 0 and opstr == '-' and not reverseorder: 
1006                  opstr = '+' 
1007                  otherstr = str(-other) 
1008              else: 
1009                  otherstr = str(other) 
1010              otherbraces = 0 
1011          else: 
1012              raise TypeError("Invalid type to combine with QuantSpec: %s"%type(other)) 
1013          if otherstr == "": 
1014              combinestr = "" 
1015              selfbraces = 0 
1016          else: 
1017              selfbraces = int((opstr == '*' or opstr == '-') and self.isCompound(['+','-']) \ 
1018                            or opstr in ['-','/'] and self.isCompound()) 
1019              combinestr = " " + opstr + " " 
1020          if not include_self: 
1021              selfstr = "" 
1022              combinestr = "" 
1023              otherbraces = 0   # override 
1024          else: 
1025              selfstr = selfbraces*"(" + self_specStr_temp + selfbraces*")" 
1026          if reverseorder: 
1027              resultstr = otherbraces*"(" + otherstr + otherbraces*")" \ 
1028                     + combinestr + selfstr 
1029          else: 
1030              resultstr = selfstr + combinestr + otherbraces*"(" + otherstr \ 
1031                                                    + otherbraces*")" 
1032          return QuantSpec("__result__", resultstr, specType)

1033   


1034 -    def __add__(self, other):


1035          return self.__combine(other, "+")

1036   


1037 -    def __mul__(self, other):


1038          return self.__combine(other, "*")

1039   


1040 -    def __sub__(self, other):


1041          return self.__combine(other, "-")

1042   


1043 -    def __div__(self, other):


1044          return self.__combine(other, "/")

1045   


1046 -    def __radd__(self, other):


1047          return self.__combine(other, "+", 1)

1048   


1049 -    def __rmul__(self, other):


1050          return self.__combine(other, "*", 1)

1051   


1052 -    def __rsub__(self, other):


1053          return self.__combine(other, "-", 1)

1054   


1055 -    def __rdiv__(self, other):


1056          return self.__combine(other, "/", 1)

1057   
1058      __truediv__ = __div__ 
1059      __rtruediv__ = __rdiv__ 
1060   


1061 -    def __rpow__(self, other):


1062          return self.__pow__(other, 1)

1063   


1064 -    def __pow__(self, other, reverseorder=0):


1065          if self.isDefined(): 
1066              selfstr = self.specStr 
1067          else: 
1068              selfstr = self.subjectToken 
1069          if reverseorder: 
1070              specType = self.specType 
1071              # other can only be non-Quant for rpow to be called 
1072              if isinstance(other, str): 
1073                  try: 
1074                      otherval = float(other) 
1075                      if otherval == 0: 
1076                          # result is 0 
1077                          return QuantSpec("__result__", '0', specType) 
1078                      elif otherval == 1: 
1079                          # result is 1 
1080                          return QuantSpec("__result__", '1', specType) 
1081                  except ValueError: 
1082                      otherstr = other 
1083              elif isinstance(other, _num_types): 
1084                  if other == 0: 
1085                      # result is 0 
1086                      return QuantSpec("__result__", '0', specType) 
1087                  elif other == 1: 
1088                      # result is 1 
1089                      return QuantSpec("__result__", '1', specType) 
1090                  else: 
1091                      otherstr = str(other) 
1092              else: 
1093                  raise TypeError("Invalid type to combine with QuantSpec: %s"%type(other)) 
1094              arg1str = otherstr 
1095              arg2str = selfstr 
1096          else: 
1097              if isinstance(other, Quantity): 
1098                  otherstr = other.name 
1099                  specType = resolveSpecTypeCombos(self.specType, other.specType) 
1100              elif isinstance(other, QuantSpec): 
1101                  specType = resolveSpecTypeCombos(self.specType, other.specType) 
1102                  # get QuantSpec's definition, if it exists 
1103                  otherstr = other() 
1104                  try: 
1105                      otherval = float(otherstr) 
1106                      if otherval == 0: 
1107                          # result is 1 
1108                          return QuantSpec("__result__", '1', specType) 
1109                      elif otherval == 1: 
1110                          # result is self 
1111                          return QuantSpec("__result__", selfstr, specType) 
1112                  except ValueError: 
1113                      # take no action 
1114                      pass 
1115              elif isinstance(other, str): 
1116                  try: 
1117                      otherval = float(other) 
1118                      if otherval == 0: 
1119                          # result is 1 
1120                          return QuantSpec("__result__", '1', specType) 
1121                      elif otherval == 1: 
1122                          # result is self 
1123                          return QuantSpec("__result__", selfstr, specType) 
1124                  except ValueError: 
1125                      otherstr = other 
1126                  specType = self.specType 
1127              elif isinstance(other, _num_types): 
1128                  specType = self.specType 
1129                  if other == 0: 
1130                      # result is 1 
1131                      return QuantSpec("__result__", '1', specType) 
1132                  elif other == 1: 
1133                      # result is self 
1134                      return QuantSpec("__result__", selfstr, specType) 
1135                  else: 
1136                      otherstr = str(other) 
1137              else: 
1138                  raise TypeError("Invalid type to combine with QuantSpec: %s"%type(other)) 
1139              arg1str = selfstr 
1140              arg2str = otherstr 
1141          return QuantSpec("__result__", 'Pow('+arg1str+','+arg2str+')', specType)

1142   


1143 -    def __neg__(self):


1144          if self.isCompound(): 
1145              return QuantSpec("__result__", "-("+self.specStr+")", self.specType) 
1146          elif self.specStr != "": 
1147              if self.specStr[0]=='-': 
1148                  return QuantSpec("__result__", self.specStr[1:], self.specType) 
1149              else: 
1150                  return QuantSpec("__result__", "-"+self.specStr, self.specType) 
1151          else: 
1152              return QuantSpec("__result__", "-"+self.subjectToken, self.specType)

1153   


1154 -    def __pos__(self):


1155          return copy(self)

1156   


1157 -    def __len__(self):


1158          return len(self.specStr)

1159      # -------------- end method group 
1160   


1161 -    def __repr__(self):


1162          return "QuantSpec %s (%s)"%(self.subjectToken,self.specType)

1163   
1164      __str__ = __call__ 
1165   


1166 -    def __eq__(self, other, diff=False):


1167          results = [] 
1168          try: 
1169              results.append(type(self) == type(other)) 
1170              results.append(self.subjectToken == other.subjectToken) 
1171              results.append(self.specStr.replace(" ","") == \ 
1172                             other.specStr.replace(" ","")) 
1173              results.append(self.specType == other.specType) 
1174              results.append(self.parser() == other.parser()) 
1175          except AttributeError, err: 
1176              if diff: 
1177                  print "Type:", className(self), results 
1178                  print "  " + str(err) 
1179              return False 
1180          if diff: 
1181              print "Type:", className(self), results 
1182          return alltrue(results)

1183   
1184   


1185 -    def __ne__(self, other):


1186          return not self.__eq__(other)

1187   
1188   


1189 -    def difference(self, other):


1190          """Show differences in comparison to another QuantSpec.""" 
1191          self.__eq__(other, diff=True)

1192   
1193   


1194 -    def isvector(self):


1195          """Is the quantity spec of the form [a,b,c]""" 
1196          try: 
1197              return '['==self.specStr[0] and ']'==self.specStr[-1] 
1198          except IndexError: 
1199              return False

1200   
1201   


1202 -    def fromvector(self, ix=None):


1203          """Turn the QuantSpec's specification of a vector to a list of 
1204          the element's specifications.""" 
1205          if self.isvector(): 
1206              if self.dim > 0: 
1207                  arglist = splitargs(self.specStr[1:-1], ['(','['], [')',']']) 
1208                  res = [QuantSpec(self.subjectToken+'_%i'%i, 
1209                                   arglist[i], self.specType) \ 
1210                          for i in range(self.dim)] 
1211              else: 
1212                  res = [QuantSpec(self.subjectToken+"_0", self.specStr[1:-1], 
1213                                       self.specType)] 
1214              if ix is None: 
1215                  return res 
1216              else: 
1217                  try: 
1218                      return res[ix] 
1219                  except IndexError: 
1220                      raise IndexError("Index out of range in vector QuantSpec") 
1221          else: 
1222              raise TypeError("This QuantSpec does not specify a vector")

1223   
1224   


1225 -    def simplify(self):


1226          """Simplify expression *IN PLACE*, but do not substitute terms from 
1227          other quantities or reduce constants such as Pi to a floating 
1228          point number.""" 
1229          qtemp = self._eval(0, {}) 
1230          self.parser = qtemp.parser 
1231          self.specStr = qtemp.specStr 
1232          self.freeSymbols = qtemp.freeSymbols 
1233          self.usedSymbols = qtemp.usedSymbols

1234   
1235   


1236 -    def renderForCode(self):


1237          """Return code-ready version of spec (e.g. uncapitalize built-in 
1238          function names)""" 
1239          return self._eval(-1, {})

1240   
1241   


1242 -    def eval(self, *scopearg, **defs):


1243          """Evaluate expression, in an optional scope dictionary (defaults to locals). 
1244          Explicit definitions override scope dictionary definitions. 
1245   
1246          Scope dictionary maps variable name -> defining object.""" 
1247          return self._eval(1, *scopearg, **defs)

1248   
1249   


1250 -    def _eval(self, eval_type, *scopearg, **defs):


1251          """Internal helper function for eval. The use of indirection using 
1252          _eval actually allows the correct scope to be accessed by whoQ 
1253          when this function automatically collects definitions.""" 
1254          # only one of defs or scopearg can be defined. 
1255          # if both are undefined then caller's local scope used to find 
1256          # possible definitions. 
1257          scope_funs = {} 
1258          if eval_type == -1: 
1259              scopeobjs = {} 
1260              scope = {} 
1261          else: 
1262              if defs == {}: 
1263                  # defs was not defined 
1264                  if scopearg == (): 
1265                      scope = {} 
1266                      # empty variable, parameter declarations pass through 
1267                      if self.specStr == '': 
1268                          scopeobjs = {} 
1269                      else: 
1270                          # try eval'ing to a number 
1271                          try: 
1272                              strval = repr(eval(self.specStr,{},{})) 
1273                          except: 
1274                              # use local names if eval_type==1 
1275                              if eval_type==1: 
1276                                  scopeobjs = whoQ() 
1277                              else: 
1278                                  scopeobjs = {} 
1279                          else: 
1280                              if self.subjectToken != "__result__": 
1281                                  scope[self.subjectToken] = strval 
1282                              scopeobjs = {} 
1283                      # process scope objects 
1284                      for key, obj in scopeobjs.iteritems(): 
1285                          if isinstance(obj, Quantity): 
1286                              if obj.name != self.subjectToken: 
1287                                  strval = str(obj.spec._eval(eval_type, {})) 
1288                                  if obj.name in [strval, "__result__"]: 
1289                                      # not a valid key 
1290                                      continue 
1291                                  # try to evaluate to float if not one already 
1292                                  try: 
1293                                      strval = repr(eval(strval,{},{})) 
1294                                  except: 
1295                                      pass 
1296                                  valq = QuantSpec('__dummy__', strval) 
1297                                  if isinstance(obj, Fun): 
1298                                      # functions dealt with in scope_funs 
1299                                      scope_funs[obj.name] = obj 
1300                                  else: 
1301                                      if valq.isCompound() and strval[0] != '(': 
1302                                          scope[obj.name] = '('+strval+')' 
1303                                      else: 
1304                                          scope[obj.name] = strval 
1305                          else: 
1306                              if obj.subjectToken != self.subjectToken: 
1307                                  strval = str(obj._eval(eval_type, {})) 
1308                                  if obj.subjectToken in [strval, "__result__"]: 
1309                                      continue 
1310                                  # try to evaluate to float if not one already 
1311                                  try: 
1312                                      strval = repr(eval(strval,{},{})) 
1313                                  except: 
1314                                      pass 
1315                                  valq = QuantSpec('__dummy__', strval) 
1316                                  if valq.isCompound() and strval[0] != '(': 
1317                                      scope[obj.subjectToken] = '('+strval+')' 
1318                                  else: 
1319                                      scope[obj.subjectToken] = strval 
1320                  else: 
1321                      if not hasattr(scopearg[0], 'keys') and len(scopearg)==1: 
1322                          raise TypeError("scope argument must be a dictionary or Point") 
1323                      scopeobjs = scopearg[0] 
1324                      # process scope objects, avoiding identity mappings of 
1325                      # tokens that could lead to infinite loops 
1326                      scope = {} 
1327                      for key, obj in scopeobjs.iteritems(): 
1328                          if key == '__result__': 
1329                              # not a valid key 
1330                              continue 
1331                          if isinstance(obj, Quantity): 
1332                              if obj.name != self.subjectToken and \ 
1333                                          (obj.name != key or obj.isspecdefined()): 
1334                                  strval = str(obj.spec._eval(eval_type, 
1335                                                  filteredDict(scopeobjs, [key], True))) 
1336                                  # try to evaluate to float if not one already 
1337                                  try: 
1338                                      strval = repr(eval(strval,{},{})) 
1339                                  except: 
1340                                      pass 
1341                                  valq = QuantSpec('__dummy__', strval) 
1342                                  if valq.isCompound() and strval[0] != '(': 
1343                                      scope[key] = '('+strval+')' 
1344                                  else: 
1345                                      scope[key] = strval 
1346                          elif isinstance(obj, QuantSpec): 
1347                              if obj.subjectToken != self.subjectToken \ 
1348                                         and (obj.subjectToken != key or obj.isspecdefined()): 
1349                                  strval = str(obj._eval(eval_type, 
1350                                                  filteredDict(scopeobjs, [key], True))) 
1351                                  # try to evaluate to float if not one already 
1352                                  try: 
1353                                      strval = repr(eval(strval,{},{})) 
1354                                  except: 
1355                                      pass 
1356                                  valq = QuantSpec('__dummy__', strval) 
1357                                  if valq.isCompound() and strval[0] != '(': 
1358                                      scope[key] = '('+strval+')' 
1359                                  else: 
1360                                      scope[key] = strval 
1361                          else: 
1362                              if isinstance(obj, _num_types): 
1363                                  if key != repr(obj): 
1364                                      scope[key] = repr(obj) 
1365                              elif isinstance(obj, str): 
1366                                  if key != obj: 
1367                                      scope[key] = obj 
1368                              else: 
1369                                  raise TypeError("Invalid scope value") 
1370              else: 
1371                  # defs was defined 
1372                  if scopearg == (): 
1373                      scopeobjs = copy(defs) 
1374                      # process scope objects 
1375                      scope = {} 
1376                      for key, obj in scopeobjs.iteritems(): 
1377                          if key == '__result__': 
1378                              continue 
1379                          if isinstance(obj, Quantity): 
1380                              if obj.name != self.subjectToken and \ 
1381                                            obj.name != key: 
1382                                  strval = str(obj.spec._eval(eval_type, 
1383                                              filteredDict(scopeobjs, 
1384                                                           [self.subjectToken], True))) 
1385                                  # try to evaluate to float if not one already 
1386                                  try: 
1387                                      strval = repr(eval(strval,{},{})) 
1388                                  except: 
1389                                      pass 
1390                                  valq = QuantSpec('__dummy__', strval) 
1391                                  if valq.isCompound() and strval[0] != '(': 
1392                                      scope[key] = '('+strval+')' 
1393                                  else: 
1394                                      scope[key] = strval 
1395                          elif isinstance(obj, QuantSpec): 
1396                              if obj.subjectToken != self.subjectToken and \ 
1397                                         obj.subjectToken != key: 
1398                                  strval = str(obj._eval(eval_type, 
1399                                              filteredDict(scopeobjs, 
1400                                                           [self.subjectToken], True))) 
1401                                  # try to evaluate to float if not one already 
1402                                  try: 
1403                                      strval = repr(eval(strval,{},{})) 
1404                                  except: 
1405                                      pass 
1406                                  valq = QuantSpec('__dummy__', strval) 
1407                                  if valq.isCompound() and strval[0] != '(': 
1408                                      scope[key] = '('+strval+')' 
1409                                  else: 
1410                                      scope[key] = strval 
1411                          else: 
1412                              if isinstance(obj, _num_types): 
1413                                  if key != repr(obj): 
1414                                      scope[key] = repr(obj) 
1415                              elif isinstance(obj, str): 
1416                                  if key != obj: 
1417                                      scope[key] = obj 
1418                              else: 
1419                                  raise TypeError("Invalid scope value") 
1420                  else: 
1421                      raise ValueError("Cannot provide both a scope dictionary and explicit " 
1422                                       "definitions") 
1423          if self.isvector(): 
1424              res_eval = [qt._eval(eval_type, scope) for qt in self.fromvector()] 
1425              return QuantSpec(self.subjectToken, 
1426                               '['+','.join([r() for r in res_eval])+']', 
1427                               self.specType) 
1428          # apply definitions in scope 
1429          arglist = scope.keys() 
1430          arglist.sort() 
1431          # convert any numeric substitutions into strings 
1432          for k, v in defs.iteritems(): 
1433              if not isinstance(v, str) and k not in [str(v), "__result__"]: 
1434                  scope[k] = str(v) 
1435              else: 
1436                  continue 
1437              valq = QuantSpec('__dummy__', scope[k])  # not necessarily = v! 
1438              # ensure brace around compound args after substitution 
1439              if valq.isCompound() and scope[k][0] != '(': 
1440                  scope[k] = '(' + scope[k] + ')' 
1441          # just ignore extra evaluation arguments for symbols that are 
1442          # not used in the expression -- e.g. for when subs() is called 
1443          # blindly, including unused pars. this saves preparing 
1444          # lists of which pars go with which expressions. 
1445          # i.e. keep following statement commented out! 
1446  ##        assert remain(arglist, freesymbs) == [], "Invalid arguments to eval" 
1447          argmap = symbolMapClass(scope) 
1448          toks = self.parser.tokenized 
1449          if toks == []: 
1450              return self 
1451          elif len(toks)==1: 
1452              mappedval=argmap(argmap(toks[0])) 
1453              if mappedval in ['E', 'Pi']: 
1454                  if eval_type==1: 
1455                      # these two have numeric values 
1456                      mappedval = repr(eval(mappedval.lower())) 
1457                  elif eval_type==-1: 
1458                      mappedval = mappedval.lower() 
1459              return QuantSpec(self.subjectToken, mappedval, self.specType) 
1460          # deal with string literals that would otherwise lose their quote marks 
1461          # during later evaluation. 
1462          # ... first, convert all single quotes to double quotes 
1463          for q_ix in range(len(toks)): 
1464              if toks[q_ix] == "'": 
1465                  toks[q_ix] = '"' 
1466          # ensure no string literals get converted 
1467          num_quotes = toks.count('"') 
1468          literals = [] 
1469          repliterals = [] 
1470          # Don't want to return math names to title case if eval_type==1 or -1 
1471          # so don't add these to the inverse map in those cases 
1472          if eval_type == 0: 
1473              FScompat_namemap = {} 
1474              FScompat_namemapInv = invertMap(mathNameMap) 
1475          elif eval_type == 1: 
1476              FScompat_namemap = mathNameMap 
1477              FScompat_namemapInv = {} 
1478          else: 
1479              # eval_type == -1 
1480              FScompat_namemap = mathNameMap 
1481              FScompat_namemapInv = {} 
1482          if num_quotes > 0: 
1483              new_toks = [] 
1484              n_ix = -1 
1485              if mod(num_quotes,2)!=0: 
1486                  raise PyDSTool_ValueError("Mismatch in number of quotes in spec") 
1487              # ensure single quotes always end up as double quotes (makes it C-compatible 
1488              #  and simplify_str() annoyingly converts doubles to singles) 
1489              FScompat_namemapInv.update({"'": '"'}) 
1490              for qname in range(int(num_quotes/2)): 
1491                  next_ix = toks[n_ix+1:].index('"') 
1492                  new_toks.extend(toks[n_ix+1:next_ix]) 
1493                  n_ix = next_ix 
1494                  end_ix = toks[n_ix+1:].index('"')+n_ix 
1495                  literal = "".join(toks[n_ix+1:end_ix+1]) 
1496                  literals.append(literal) 
1497                  qlit = '"'+literal+'"' 
1498                  replit = '"__literal_'+literal+'"' 
1499                  repliterals.append(replit[1:-1]) 
1500                  new_toks.append(replit[1:-1]) 
1501                  # add to inverse name map to restore literals after eval() 
1502                  # (don't include quotes) 
1503                  FScompat_namemapInv[replit[1:-1]] = qlit 
1504              # finish adding the remaining tokens 
1505              new_toks.extend(toks[end_ix+2:]) 
1506              qtemp = QuantSpec(self.subjectToken, "".join(argmap(new_toks)), 
1507                            self.specType) 
1508          else: 
1509              new_toks = toks 
1510              qtemp = self.__copy__() 
1511              qtemp.mapNames(argmap) 
1512          # now apply scope mapping so that literals remain unaffected. 
1513          # second pass for one level of indirect references 
1514          # but only use keys that weren't present before, to avoid circular 
1515          # mapping: e.g. s->t then t->s 
1516          if scope != {}: 
1517              qtemp.mapNames(filteredDict(scope,remain(toks,scope.keys()))) 
1518          # references to builtin aux functions or macros require temp 
1519          # definitions of those as Fun objects 
1520          qtoks = qtemp.parser.tokenized 
1521          localfuncs = intersect(_localfuncnames, qtoks) 
1522          for symb in remain(qtemp.freeSymbols, repliterals): 
1523              if symb not in _avoid_math_symbols: 
1524                  FScompat_namemap[symb] = symb.replace('.', '_') 
1525                  FScompat_namemapInv[FScompat_namemap[symb]] = symb 
1526          for symb in localfuncs: 
1527              FScompat_namemap[symb] = symb+"_" 
1528              FScompat_namemapInv[symb+"_"] = symb 
1529          qtemp.mapNames(FScompat_namemap) 
1530          qtemp_feval = copy(qtemp) 
1531          # map these separately because need to keep the two dicts distinct 
1532          qtemp_feval.mapNames(feval_map_const) 
1533          qtemp_feval.mapNames(feval_map_symb) 
1534          # once math names -> lower case, try mapping scope defs again 
1535          qtemp_feval.mapNames(scope) 
1536          # are qtemp or qtemp_feval already containing only numeric values 
1537          # or built-in math functions? 
1538          num_qtemp = qtemp.freeSymbols == [] 
1539          num_qtemp_feval = qtemp_feval.freeSymbols == [] 
1540          sq = str(qtemp) 
1541          sq_feval = str(qtemp_feval) 
1542          # try pre-simpilfying the expressions 
1543          try: 
1544              defstr = simplify_str(sq) 
1545          except (SyntaxError, MemoryError): 
1546              # Probably from 'for' syntax, which contains a clause with 
1547              # a '+' or '-' symbol without being part of an arithmetical expr 
1548              # Would like to make this cleaner... 
1549              defstr = sq 
1550          except TypeError: 
1551              print self.specStr, sq 
1552              raise 
1553          else: 
1554              # HACK: fix simplify_str()'s return of vector arg with round braces 
1555              if self.isvector() and defstr[0]=='(' and defstr[-1]==')': 
1556                  defstr = '['+defstr[1:-1]+']' 
1557          try: 
1558              defstr_feval = simplify_str(sq_feval) 
1559          except (SyntaxError, MemoryError): 
1560              # Probably from 'for' syntax, which contains a clause with 
1561              # a '+' or '-' symbol without being part of an arithmetical expr 
1562              # Would like to make this cleaner... 
1563              defstr_feval = sq_feval 
1564          else: 
1565              # HACK: fix simplify_str()'s return of vector arg with round braces 
1566              if self.isvector() and defstr_feval[0]=='(' and defstr_feval[-1]==')': 
1567                  defstr_feval = '['+defstr_feval[1:-1]+']' 
1568          # otherwise, continue pre-process simplified definition strings 
1569          for clause_type in ('if', 'max', 'min', 'sum'): 
1570              if clause_type not in localfuncs: 
1571                  continue 
1572              # protect condition or max/min clauses from being Python-eval'd 
1573              # because Python will turn it into a boolean value or mess up max/min! 
1574              # ... so substitute the whole clause with a dummy name 
1575              num_stmts = qtoks.count(clause_type) 
1576              n_ix = 0 
1577              for stmt_ix in range(num_stmts): 
1578                  n_ix = qtoks[n_ix:].index(clause_type) + n_ix 
1579                  if clause_type == 'if': 
1580                      stop_ix = qtoks[n_ix:].index(',')+n_ix 
1581                  else: 
1582                      stop_ix = findEndBrace(qtoks[n_ix+1:])+n_ix+1 
1583                  clause_original = "".join(qtoks[n_ix+2:stop_ix]) 
1584                  clause = "".join(qtemp.parser.tokenized[n_ix+2:stop_ix]) 
1585                  defstr = defstr.replace(clause,'__temp_clause__'+str(stmt_ix)) 
1586                  defstr_feval = defstr_feval.replace(clause,'__temp_clause__'+str(stmt_ix)) 
1587                  # add to inverse name map to restore clauses after eval() 
1588                  FScompat_namemapInv['__temp_clause__'+str(stmt_ix)] = clause_original 
1589                  qtemp.freeSymbols.append('__temp_clause__'+str(stmt_ix)) 
1590                  n_ix = stop_ix 
1591          else: 
1592              num_stmts = 0 
1593          # add underscored suffix to built in function names to avoid clashing 
1594          # with actual Python names, e.g. "if", "for" 
1595          if not num_qtemp: 
1596              freenamelist = remain(qtemp.freeSymbols, 
1597                                    allmathnames_symbolic + localfuncs) 
1598              local_scope = dict(zip(freenamelist,[QuantSpec(symb) for symb in freenamelist])) 
1599              local_scope.update(local_fndef) 
1600              local_scope_feval = copy(local_scope) 
1601              if eval_type == 1: 
1602                  # Allow constants like Pi and E and function calls like 
1603                  # Log(10) to become floating point numbers 
1604                  local_scope_feval.update(math_globals) 
1605              # crucial to update with scope_funs last, so that these "true" definitions 
1606              # can overwrite the "dummy" placeholder definitions that were there 
1607              local_scope.update(scope_funs) 
1608              local_scope_feval.update(scope_funs) 
1609          else: 
1610              if eval_type == 1: 
1611                  local_scope = {} 
1612                  local_scope_feval = math_globals 
1613              else: 
1614                  local_scope = local_scope_feval = {} 
1615          # DEBUG: info(local_scope, "Local scope") 
1616          # because the free names have been defined locally as QuantSpecs, 
1617          # eval will return another QuantSpec 
1618          if eval_type == 1: 
1619              # attempt to evaluate to a numeric type, but settle for the most 
1620              # simplified numeric version otherwise (including lower case functions) 
1621              try: 
1622                  new_qtemp = eval(defstr_feval, {}, local_scope_feval) 
1623                  assert isinstance(new_qtemp, (Quantity, QuantSpec, str, list, \ 
1624                                          _num_types)) 
1625              except: 
1626                  new_qtemp = QuantSpec(self.subjectToken, defstr_feval, self.specType) 
1627          elif eval_type == 0: 
1628              try: 
1629                  new_qtemp = eval(defstr, {}, local_scope) 
1630                  assert isinstance(new_qtemp, (Quantity, QuantSpec, str, list, \ 
1631                                      _num_types)) 
1632                  # assume that number of braces correlates with complexity 
1633                  # only keep this if trying to simplify and expression becomes 
1634                  # simpler, or we're trying to reduce float sub-expressions 
1635                  assert str(new_qtemp).count('(') <= defstr.count('('), \ 
1636                         "Didn't get simpler -- going to except clause" 
1637              except: 
1638                  new_qtemp = QuantSpec(self.subjectToken, defstr, self.specType) 
1639              # if just simplifying then assume that number of braces 
1640              # correlates with complexity 
1641              if str(new_qtemp).count('(') > self.specStr.count('('): 
1642                  # didn't get simpler, so quit now 
1643                  return self 
1644          else: 
1645              new_qtemp = QuantSpec(self.subjectToken, defstr, self.specType) 
1646          if isinstance(new_qtemp, _num_types): 
1647              new_qtemp = QuantSpec(self.subjectToken, repr(new_qtemp), self.specType) 
1648          elif not isinstance(new_qtemp, QuantSpec): 
1649              # could be list of Quantities or QuantSpecs 
1650              new_qtemp = QuantSpec(self.subjectToken, strIfSeq(new_qtemp), self.specType) 
1651          else: 
1652              # Only do the following when not rendering for code output 
1653              # Turn math functions back to title case if float eval failed 
1654              # but only those that weren't originally in lower case 
1655              if eval_type != -1: 
1656                  new_qtemp.mapNames(filteredDict(inverseMathNameMap, 
1657                                                  qtoks, 1)) 
1658              # make sure specType is set correctly 
1659              new_qtemp.specType = self.specType 
1660          new_qtemp.mapNames(FScompat_namemapInv) 
1661          return new_qtemp

1662   
1663   


1664 -    def tonumeric(self):


1665          if self.freeSymbols == []: 
1666              if self.isvector(): 
1667                  try: 
1668                      return array(eval(self.specStr, {}, {})) 
1669                  except: 
1670                      raise TypeError("Cannot convert this QuantSpec to a numeric type") 
1671              else: 
1672                  try: 
1673                      return eval(self.specStr, {}, {}) 
1674                  except: 
1675                      raise TypeError("Cannot convert this QuantSpec to a numeric type") 
1676          else: 
1677              raise TypeError("Cannot convert this QuantSpec to a numeric type")

1678   
1679   


1680 -    def mapNames(self, nameMap):


1681          """Can pass a dictionary or a symbolMapClass instance to this method, 
1682          but it will convert it internally to a symbolMapClass.""" 
1683          namemap = symbolMapClass(nameMap) 
1684          newsubj = namemap(self.subjectToken) 
1685          if isinstance(newsubj, str) and \ 
1686                (isNameToken(newsubj, True) or \ 
1687                 isHierarchicalName(newsubj, treatMultiRefs=True)): 
1688              self.subjectToken = newsubj 
1689          else: 
1690              print "Attempted to rename %s with object of type %s, value:"%(self.subjectToken,str(type(newsubj))), newsubj 
1691              raise TypeError("Cannot rename a QuantSpec with a non-name string") 
1692          toks = [namemap(t) for t in self.parser.tokenized] 
1693          # strip() in case spaces were preserved and there were trailing spaces 
1694          # (these can confuse function definitions made from these into thinking 
1695          # that there's a badly indented code block following the function body) 
1696          self.specStr = "".join(toks).strip() 
1697          self.parser.specStr = self.specStr 
1698          self.specStr = \ 
1699                  self.parser.parse(self.parser.ignoreTokens, None, 
1700                                    includeProtected=self.parser.includeProtected, 
1701                                    reset=True) 
1702          self.usedSymbols = self.parser.usedSymbols 
1703          self.freeSymbols = self.parser.freeSymbols

1704   
1705   
1706      # difficult to reconstruct original options at __init__, so just copy by pickle 


1707 -    def __copy__(self):


1708          pickledself = pickle.dumps(self) 
1709          return pickle.loads(pickledself)

1710   
1711   


1712 -    def __deepcopy__(self, memo=None, _nil=[]):


1713          pickledself = pickle.dumps(self) 
1714          return pickle.loads(pickledself)

1715   
1716   
1717  # -------------------------------------------------------------------------- 
1718  ### Multi-quantity reference and multi-quantity definition utilities 
1719   


1720 -def processMultiDef(s, specstr, specType):


1721      # Assume attempt was made at using a multiple Quantity definition 
1722      # (i.e. that isMultiDef() returned true). 
1723      # so if further tests show the wrong syntax, then cause error, 
1724      # not just return False (because square brackets are not otherwise 
1725      # allowed in a QuantSpec subjectToken. 
1726      # Correct format is "<symbol 1>[<symbol 2>,<int 1>,<int 2>]" 
1727      # where s1 != s2 and i1 < i2, and s1 does not end in a number 
1728      m = parserObject(s, ignoreTokens=['[']) 
1729      i1 = eval(m.tokenized[4]) 
1730      i2 = eval(m.tokenized[6]) 
1731      rootname = m.tokenized[0] 
1732      ixname = m.tokenized[2] 
1733      if len(rootname) > 1: 
1734          test_ix = -1 
1735      else: 
1736          test_ix = 0 
1737      root_test = isNameToken(rootname) and rootname[test_ix] not in num_chars 
1738      if not alltrue([len(m.usedSymbols)==4, m.tokenized[1]=="[", 
1739                  root_test, isNameToken(ixname), 
1740                  m.tokenized[7]=="]", rootname != ixname, 
1741                  m.tokenized[3]==m.tokenized[5]==",", 
1742                  compareNumTypes(type(i1), type(i2)) and isinstance(i1, _int_types), 
1743                   i1 < i2]): 
1744          raise ValueError("Syntax error in multiple Quantity definition " 
1745                           "spec: " + s) 
1746      ## Check that all x[f(i)] references have f(i) integers 
1747      # any references outside i1..i2 become freeSymbols, for x 
1748      # either z or another symbol (known as 'root' below). 
1749      # Also, any zXX refs present in the mrefs, where XX is not in 
1750      # the range [i1,i2], are free symbols of this Quantity 
1751      # because they aren't being defined here 
1752      defrange = xrange(i1,i2+1) 
1753      # Quantity subject token is 'z[<ixname>]' 
1754      # and future 'name' attribute 
1755      token = rootname + "[" + ixname + "]" 
1756      all_mrefs = {}    # dict of rootname -> valsref'd list 
1757      mrefs = findMultiRefs(specstr) 
1758      actual_freeSymbols = [] 
1759      for tup in mrefs: 
1760          mref = processMultiRef(tup[1], tup[0], 
1761                                ixname, defrange) 
1762          all_mrefs[tup[0]] = mref 
1763          if tup[0] == rootname: 
1764              # ignore ones being defined here 
1765              out_of_range = remain(mref, defrange) 
1766          else: 
1767              out_of_range = mref 
1768          # only add those names not yet present in freeSymbols 
1769          actual_freeSymbols.extend(remain([tup[0] + str(i) for i in\ 
1770                               out_of_range], actual_freeSymbols)) 
1771      ## Find direct references to z1-z10 in spec. 
1772      # avoid using square brackets when making temp_spec 
1773      surrogateToken = "__"+rootname+"__" 
1774      surrogateSpec = specstr 
1775      for (r, targetExpr) in mrefs: 
1776          if r==rootname: 
1777              surrogateSpec = surrogateSpec.replace(targetExpr, 
1778                                                    surrogateToken) 
1779      # making temp a QuantSpec gives us a free check for the 
1780      # legality of the specType 
1781      try: 
1782          temp_qspec = QuantSpec(surrogateToken, surrogateSpec, 
1783                                 specType, treatMultiRefs=True) 
1784      except ValueError, e: 
1785          # replace temp name __z__ with actual token name z[i] 
1786          new_e = (e[0].replace(surrogateToken, token)) 
1787          raise ValueError, new_e 
1788      for f in temp_qspec.freeSymbols: 
1789          try: 
1790              is_int = f[-1] in num_chars 
1791          except: 
1792              is_int = False 
1793          if is_int: 
1794              # f ends in a number 
1795              p = findNumTailPos(f) 
1796              # find out if the root of the symbol is rootname 
1797              # then find out if the tail number is in the 
1798              # defining range. 
1799              if f[:p] == rootname and int(f[p:]) \ 
1800                      not in defrange: 
1801                  # it is not in range, so is not being defined 
1802                  # by this multiple Quantity definition, thus 
1803                  # it is a true free Symbol of this Quantity 
1804                  if f not in actual_freeSymbols: 
1805                      actual_freeSymbols.append(f) 
1806          elif f == surrogateToken or f == ixname: 
1807              pass 
1808          else: 
1809              # f is a free symbol unrelated to token 
1810              if f not in actual_freeSymbols: 
1811                  actual_freeSymbols.append(f) 
1812      # use multi-ref treatment option for QuantSpec 
1813      # NOTE -- 'subjectToken' was just 'token' 
1814      subjectToken = rootname+'['+ixname+','+str(i1)+','+str(i2)+']' 
1815      actual_spec = QuantSpec(subjectToken, specstr, specType, 
1816                              treatMultiRefs=True) 
1817      mdef = (rootname, ixname, i1, i2, token) 
1818      return mdef, actual_spec, actual_freeSymbols

1819   
1820   


1821 -def isMultiDef(s):


1822      """Determine whether string is a candidate multiple Quantity definition.""" 
1823   
1824      temp = parserObject(s, ignoreTokens=['[']) 
1825      try: 
1826          return alltrue([not temp.isCompound(), len(temp.tokenized)==8, 
1827                          isNameToken(temp.tokenized[0]), 
1828                          isNameToken(temp.tokenized[2]), 
1829                          temp.tokenized[1]=="[", temp.tokenized[-1]=="]"]) 
1830      except IndexError: 
1831          # e.g. if string doesn't contain enough tokens 
1832          return False

1833   
1834   


1835 -def findMultiRefs(s):


1836      temp = parserObject(s, ignoreTokens=['[']) 
1837      # reflist contains tuples of (rootname, expr) 
1838      reflist = [] 
1839      searchSymbols = temp.freeSymbols 
1840      if len(temp.freeSymbols) == 0: 
1841          return reflist 
1842      # for each free symbol, see if it's followed by [ ... ] 
1843      toks_to_process = temp.tokenized 
1844      while True: 
1845          next_ixs = [] 
1846          for f in searchSymbols: 
1847              try: 
1848                  next_ixs.append(toks_to_process.index(f)) 
1849              except ValueError: 
1850                  # not found 
1851                  pass 
1852          if len(next_ixs) > 0: 
1853              next_free = min(next_ixs) 
1854          else: 
1855              break 
1856          # find contents of [ ... ] 
1857          try: 
1858              if toks_to_process[next_free+1] == "[": 
1859                  try: 
1860                      rb_ix = toks_to_process[next_free+1:].index("]") \ 
1861                              + next_free + 1 
1862                  except ValueError: 
1863                      raise ValueError("Mismatched square braces in multiple " 
1864                                       "quantity reference specification: " + s) 
1865                  reflist.append((toks_to_process[next_free], 
1866                             "".join(toks_to_process[next_free:rb_ix+1]))) 
1867                  toks_to_process = toks_to_process[rb_ix+1:] 
1868              else: 
1869                  toks_to_process = toks_to_process[next_free+1:] 
1870          except IndexError: 
1871              # symbol is last in tokenized 
1872              break 
1873      return reflist

1874   
1875   


1876 -def isMultiRef(s):


1877      """Simple litmus test for candidate multiple quantity reference 
1878      (or definition). 
1879   
1880      Requires further testing using one of the other associated multiple 
1881      quantity reference/definition tests.""" 
1882      if len(s) > 1: 
1883          sparts = s.split('[') 
1884          # check that '[' occurs as '<name>[' 
1885          if len(sparts) > 1: 
1886              try: 
1887                  reftest = alltrue([sp[-1].isalnum() for sp in sparts[:-1]]) 
1888              except IndexError: 
1889                  # e.g. sp == '' 
1890                  reftest = False 
1891          else: 
1892              reftest = False 
1893          return reftest 
1894      else: 
1895          return False

1896   
1897   


1898 -def processMultiRef(s, rootname, ixname, ok_range):


1899      # Is valid multiple quantity ref? ... correct format is 
1900      # "rootname[<expr in ixname>]" for a valid rootname (does not end in 
1901      # a number), where the expression evaluates to an integer when ixname 
1902      # is substituted by an integer in the range ok_range = [i1,i2]. 
1903      temp = parserObject(s, ignoreTokens=['[']) 
1904      try: 
1905          if len(rootname) > 1: 
1906              test_ix = -1 
1907          else: 
1908              test_ix = 0 
1909          roottest = rootname[test_ix] not in num_chars 
1910          if not alltrue([roottest, len(temp.tokenized) >= 4, 
1911                      temp.tokenized[0] == rootname, 
1912                      rootname in temp.freeSymbols, 
1913                      ixname in temp.freeSymbols, 
1914                      rootname not in temp.tokenized[1:], 
1915                      temp.tokenized[1]=="[", temp.tokenized[-1]=="]"]): 
1916              raise ValueError("Error in multiple Quantity reference " 
1917                               "spec: " + s) 
1918          # then we have a fully syntax-checked multiple quantity ref 
1919      except IndexError: 
1920          # e.g. temp.tokenized[1] --> index out of bounds 
1921          raise ValueError("Error in multiple Quantity reference " 
1922                           "spec: " + s) 
1923      # test evaluation of every expression for ixname in range [i1, i2] to be 
1924      # an integer, where expr is everything between the square brackets 
1925      original_tokenlist = temp.tokenized[2:-1] 
1926      ixname_occs = [] 
1927      for i in xrange(len(original_tokenlist)): 
1928          if original_tokenlist[i] == ixname: 
1929              ixname_occs.append(i) 
1930      # make a list of all referenced integers by this expression, over this 
1931      # range ok_range = [i1,i2] 
1932      vals_refd = [] 
1933      try: 
1934          for ixval in ok_range: 
1935              tokenlist = original_tokenlist 
1936              for i in ixname_occs: 
1937                  tokenlist[i] = str(ixval) 
1938              expr = "".join(tokenlist) 
1939              try: 
1940                  val = eval(expr) 
1941              except: 
1942                  val = None 
1943              if not isinstance(val, _int_types): 
1944                  if int(val) == val: 
1945                      # functions like scipy.mod return floats even 
1946                      # though their values are equal to integers! 
1947                      val = int(val) 
1948                  else: 
1949                      raise AssertionError 
1950              if val not in vals_refd: 
1951                  vals_refd.append(val) 
1952      except AssertionError: 
1953          raise ValueError("Non-integer evaluation of the index-generating " 
1954                           "expression of multiple Quantity reference spec:" 
1955                           " " + s) 
1956      return vals_refd

1957   
1958   


1959 -def isMultiDefClash(obj, multiDefRefs, parentname=None):


1960      result = False    # default, initial value 
1961      if isMultiDef(obj.name): 
1962          objinfo = obj.multiDefInfo 
1963          # objinfo == (True, rootname, ixname, i1, i2) 
1964          if objinfo[0]: 
1965              if parentname is None: 
1966                  rootname = objinfo[1] 
1967              else: 
1968                  rootname = parentname + NAMESEP + objinfo[1] 
1969              try: 
1970                  infotuple = multiDefRefs[rootname] 
1971                  # infotuple == (ixname, i1, i2) 
1972                  is_present = True 
1973              except KeyError: 
1974                  is_present = False 
1975              if is_present: 
1976                  # have more work to do, to establish which numbers are 
1977                  # already defined. 
1978                  obj_range = Interval('obj', int, 
1979                                       [objinfo[3], objinfo[4]]) 
1980                  self_range = Interval('self', int, 
1981                                        [infotuple[1], infotuple[2]]) 
1982                  if obj_range.intersect(self_range) is not None: 
1983                      result = True 
1984          else: 
1985              # obj.name should not have returned as isMultiDef! 
1986              raise ValueError("Object %s contains invalid multiple quantity" 
1987                               " definitions"%obj.name) 
1988      else: 
1989          # is the single variable obj.name one of the multiDefRefs 
1990          try: 
1991              p = findNumTailPos(obj.name) 
1992          except ValueError: 
1993              # obj.name does not end in a number, so cannot clash 
1994              return False 
1995          obj_rootname = obj.name[:p] 
1996          obj_ix = int(obj.name[p:])    # known to be ok integer 
1997          for rootname, infotuple in multiDefRefs.iteritems(): 
1998              if obj_rootname == rootname: 
1999                  # root of the name is present 
2000                  # so check if the number falls in the subject range 
2001                  if obj_ix in xrange(infotuple[3], infotuple[4]+1): 
2002                      result = True 
2003                      break 
2004      return result

2005   
2006   


2007 -def evalMultiRefToken(mref, ixname, val):


2008      """Helper function for evaluating multi-reference tokens for 
2009      given index values.""" 
2010      return eval(mref.replace(ixname,str(val)), {}, {})

2011   
2012  # ------------------------------------------------------------------------ 
2013   


2014 -class Quantity(object):


2015      """Abstract class for symbolic numerical quantities.""" 
2016   
2017      typestr = None 
2018      compatibleGens = () 
2019      targetLangs = () 
2020   


2021 -    def __init__(self, spec, name="", domain=None, specType=None):


2022          self.multiDefInfo = (False, "", "", 0, 0)   # initial, default value 
2023          if isinstance(strIfSeq(spec), str): 
2024              spec = strIfSeq(spec) 
2025              if isNameToken(spec): 
2026                  # then we're declaring a single symbol 
2027                  if isNameToken(name): 
2028                      # spec symbol is being renamed to name 
2029                      token = name.strip() 
2030                      actual_spec = QuantSpec(token, spec, specType) 
2031                  elif name=="": 
2032                      # spec and name are to be the same 
2033                      token = spec.strip() 
2034                      actual_spec = QuantSpec(token, "", specType) 
2035                  else: 
2036                      raise ValueError("name argument contains illegal symbols") 
2037              elif isNameToken(name): 
2038                  # declaring a symbol and its non-trivial definition in spec 
2039                  token = name.strip() 
2040                  actual_spec = QuantSpec(token, spec, specType) 
2041              elif isMultiDef(name): 
2042                  # declaring multiple symbols at once, with their definition 
2043                  # in spec 
2044                  if self.typestr == 'auxfn': 
2045                      raise TypeError("Multiple quantity definition is invalid " 
2046                                      "for function definitions") 
2047                  mdef, actual_spec, actual_freeSymbols = processMultiDef(name, 
2048                                                                          spec, 
2049                                                                      specType) 
2050                  # z[i,1,10] --> rootname = 'z', ixname = 'i', i1=1, i2=10 
2051                  rootname = mdef[0] 
2052                  ixname = mdef[1] 
2053                  i1 = mdef[2] 
2054                  i2 = mdef[3] 
2055                  token = name 
2056                  self.multiDefInfo = (True, rootname, ixname, i1, i2) 
2057              else: 
2058                  print "Token name argument was:", name 
2059                  raise ValueError("Invalid Quantity token name") 
2060          elif isinstance(spec, QuantSpec): 
2061              actual_spec = copy(spec) 
2062              if spec.subjectToken == "__result__" or name != "": 
2063                  if isNameToken(name): 
2064                      token = name.strip() 
2065                      actual_spec.subjectToken = token 
2066                  elif isMultiDef(name): 
2067                      # declaring multiple symbols at once, with their definition 
2068                      # in spec 
2069                      if self.typestr == 'auxfn': 
2070                          raise TypeError("Multiple quantity definition is invalid " 
2071                                          "for function definitions") 
2072                      if specType is not None: 
2073                          actual_specType = specType 
2074                      else: 
2075                          actual_specType = spec.specType 
2076                      mdef, actual_spec, actual_freeSymbols = \ 
2077                             processMultiDef(name, spec.specStr, actual_specType) 
2078                      # z[i,1,10] --> rootname = 'z', ixname = 'i', i1=1, i2=10 
2079                      rootname = mdef[0] 
2080                      ixname = mdef[1] 
2081                      i1 = mdef[2] 
2082                      i2 = mdef[3] 
2083                      token = name 
2084                      self.multiDefInfo = (True, rootname, ixname, i1, i2) 
2085                  else: 
2086                      print "Token name argument was:", name 
2087                      raise TypeError("Invalid type for Quantity token name") 
2088              else: 
2089                  token = spec.subjectToken 
2090              if specType is not None: 
2091                  # override default specType from QuantSpec in case 
2092                  # e.g. it was made from connecting RHSfuncSpec with 
2093                  # something --> RHSfuncSpec, but user wants this Quantity 
2094                  # to be a different type 
2095                  actual_spec.specType = specType 
2096          elif isinstance(spec, (Par, Var)): 
2097              # referring to a parameter or variable name as a new variable 
2098              if isNameToken(name): 
2099                  token = name.strip() 
2100              else: 
2101                  raise ValueError("Invalid name '%s' for this Quantity"%name) 
2102              actual_spec = QuantSpec(token, spec.name) 
2103              if specType is not None: 
2104                  # override default specType from QuantSpec in case 
2105                  # e.g. it was made from connecting RHSfuncSpec with 
2106                  # something --> RHSfuncSpec, but user wants this Quantity 
2107                  # to be a different type 
2108                  actual_spec.specType = specType 
2109          elif isinstance(spec, _num_types): 
2110              # convert numeric literals 
2111              token = name.strip() 
2112              actual_spec = QuantSpec(token, repr(spec), specType) 
2113          else: 
2114              raise TypeError("Invalid spec argument") 
2115          if token == 'for': 
2116              raise ValueError("Cannot use reserved macro name 'for' as a " 
2117                               "Quantity's name") 
2118          else: 
2119              self.name = token 
2120          self.spec = actual_spec 
2121          self.specType = self.spec.specType 
2122          if self.multiDefInfo[0]: 
2123              # must override spec's free symbol list with doctored one 
2124              self.freeSymbols = remain(actual_freeSymbols, self.name) 
2125  #            self.usedSymbols = actual_usedSymbols 
2126          else: 
2127              self.freeSymbols = remain(self.spec.freeSymbols, self.name) 
2128          self.usedSymbols = self.spec.usedSymbols 
2129          # various ways to specify domain ... 
2130          if domain is None: 
2131              # assume continuous-valued from -Inf to Inf 
2132              self.domain = (float, Continuous, [-Inf, Inf]) 
2133          else: 
2134              self.setDomain(domain) 
2135          try: 
2136              # assume max of rank 2 
2137              self.dim = getdim(self.spec.specStr)  # for vector 
2138          except IndexError: 
2139              # spec not long enough therefore self cannot be a vector 
2140              self.dim = 0 
2141          self.funcSpecDict = {} 
2142          self.validate()

2143   
2144   


2145 -    def setDomain(self, domain):


2146          if isinstance(domain, list): 
2147              # assume continuous-valued float 
2148              assert len(domain)==2, "Domain-as-list must have length 2" 
2149              d = (float, Continuous, domain) 
2150          elif isinstance(domain, tuple): 
2151              # full spec is a 3-tuple 
2152              assert len(domain)==3, "Domain-as-tuple must have length 3" 
2153              assert compareNumTypes(domain[0], _num_types), \ 
2154                     "Invalid domain numeric type" 
2155              assert domain[1] in [Continuous, Discrete], \ 
2156                              "Invalid domain type" 
2157              if compareNumTypes(domain[0], _int_types): 
2158                  assert domain[1] is Discrete, "Domain type mismatch" 
2159              d = domain 
2160          else: 
2161              raise TypeError("Invalid domain argument: "+type(domain)) 
2162          self.domain = d

2163   


2164 -    def redefine(self, specStr):


2165          self.spec.redefine(specStr)

2166   


2167 -    def isspecdefined(self):


2168          return self.spec.specStr.strip() != ''

2169   
2170   


2171 -    def __contains__(self, tok):


2172          return tok in self.spec.parser.tokenized

2173   
2174   


2175 -    def __call__(self):


2176          return copy(self.spec)

2177   


2178 -    def __len__(self):


2179          return self.dim

2180   


2181 -    def isvector(self):


2182          """Is the quantity spec of the form [a,b,c]""" 
2183          try: 
2184              # minimum check necessary at this point to determine this 
2185              return '['==self.spec.specStr[0] and ']'==self.spec.specStr[-1] 
2186          except IndexError: 
2187              return False

2188   
2189   


2190 -    def fromvector(self, ix=None):


2191          """Turn the Quantity's specification of a vector to a list of 
2192          the element's specifications.""" 
2193          if self.isvector(): 
2194              if self.dim > 0: 
2195                  arglist = splitargs(self.spec.specStr[1:-1], ['(','['], [')',']']) 
2196                  res = [QuantSpec(self.name+'_%i'%i, 
2197                                   arglist[i], self.specType) \ 
2198                          for i in range(self.dim)] 
2199              else: 
2200                  res = [QuantSpec(self.name+"_0", self.spec.specStr[1:-1], 
2201                                       self.specType)] 
2202              if ix is None: 
2203                  return res 
2204              else: 
2205                  try: 
2206                      return res[ix] 
2207                  except IndexError: 
2208                      raise IndexError("Index out of range in vector Quantity") 
2209          else: 
2210              raise TypeError("This Quantity does not specify a vector")

2211   
2212   


2213 -    def __setitem__(self, x, v):


2214          raise NotImplementedError

2215   


2216 -    def __delitem__(self, x):


2217          raise NotImplementedError

2218   


2219 -    def __getitem__(self, x):


2220          # kludge! This is overloaded. 
2221          # Self may be either a multiRef or for a vector 
2222          if self.multiDefInfo[0]: 
2223              m = self.multiDefInfo 
2224              rootname = m[1] 
2225              ixname = m[2] 
2226              i1 = m[3] 
2227              i2 = m[4] 
2228              if x in xrange(i1,i2+1): 
2229                  tokenized = [] 
2230                  for t in self.spec.parser.tokenized: 
2231                      # can trust that t starting with '[' is a valid mref 
2232                      # because it was checked at initialization 
2233                      if t[0] == '[': 
2234                          # append evaluated integer without brackets 
2235                          tokenized.append(str(evalMultiRefToken(t[1:-1], 
2236                                                                 ixname, x))) 
2237                      elif t == ixname: 
2238                          tokenized.append(str(x)) 
2239                      else: 
2240                          tokenized.append(t) 
2241                  # make the appropriate sub-type of Quantity to return 
2242                  q = Quantity.__new__(self.__class__) 
2243                  q.__init__("".join(tokenized), rootname + str(x), 
2244                             specType=self.specType) 
2245                  return q 
2246              else: 
2247                  raise IndexError("Index to multiple Quantity definition out " 
2248                                   "of the valid range [%i,%i]"%(i1,i2)) 
2249          elif self.isvector(): 
2250              return self.fromvector(x) 
2251          else: 
2252              raise IndexError("Quantity %s does not contain multiple " 
2253                       "definitions and is not a vector of symbols"%self.name)

2254   


2255 -    def tonumeric(self):


2256          if self.spec.freeSymbols == []: 
2257              if self.isvector(): 
2258                  try: 
2259                      return array(eval(self.spec.specStr, {}, {})) 
2260                  except: 
2261                      raise TypeError("Cannot convert this Quantity to a numeric type") 
2262              else: 
2263                  try: 
2264                      return eval(self.spec.specStr, {}, {}) 
2265                  except: 
2266                      raise TypeError("Cannot convert this Quantity to a numeric type") 
2267          else: 
2268              raise TypeError("Cannot convert this Quantity to a numeric type")

2269   
2270   
2271      # -------------- method group for spec string assembly 


2272 -    def __combine(self, other, opstr, reverseorder=0):


2273          if isinstance(other, QuantSpec): 
2274              other_specType = other.specType 
2275              otherstr = other() 
2276          elif isinstance(other, Quantity): 
2277              other_specType = other.specType 
2278              otherstr = other.name 
2279          elif isinstance(other, str): 
2280              other_specType = 'ExpFuncSpec' 
2281              otherstr = other 
2282          elif isinstance(other, _num_types): 
2283              otherstr = str(other) 
2284              other_specType = 'ExpFuncSpec' 
2285          else: 
2286              raise TypeError("Invalid types to combine") 
2287          try: 
2288              r1 = QuantSpec("__result__", otherstr, other_specType) 
2289          except TypeError: 
2290              print "Failed to compile: '" + otherstr + "'" 
2291              raise 
2292          r2 = QuantSpec("__result__", self.name, self.specType) 
2293          if reverseorder: 
2294              return eval('r1' + opstr + 'r2') 
2295          else: 
2296              return eval('r2' + opstr + 'r1')

2297   


2298 -    def __add__(self, other):


2299          return self.__combine(other, "+")

2300   


2301 -    def __mul__(self, other):


2302          return self.__combine(other, "*")

2303   


2304 -    def __sub__(self, other):


2305          return self.__combine(other, "-")

2306   


2307 -    def __div__(self, other):


2308          return self.__combine(other, "/")

2309   


2310 -    def __radd__(self, other):


2311          return self.__combine(other, "+", 1)

2312   


2313 -    def __rmul__(self, other):


2314          return self.__combine(other, "*", 1)

2315   


2316 -    def __rsub__(self, other):


2317          return self.__combine(other, "-", 1)

2318   


2319 -    def __rdiv__(self, other):


2320          return self.__combine(other, "/", 1)

2321   
2322      __truediv__ = __div__ 
2323      __rtruediv__ = __rdiv__ 
2324   


2325 -    def __pow__(self, other):


2326          dummy = QuantSpec('__result__', self.name) 
2327          return dummy.__pow__(other)

2328   


2329 -    def __rpow__(self, other):


2330          dummy = QuantSpec('__result__', self.name) 
2331          return dummy.__pow__(other, 1)

2332   


2333 -    def __neg__(self):


2334          return QuantSpec("__result__", "-" + self.name, self.specType)

2335   


2336 -    def __pos__(self):


2337          return QuantSpec("__result__", self.name, self.specType)

2338   
2339      # -------------- end method group 
2340   


2341 -    def mapNames(self, nameMap):


2342          if self.name in nameMap: 
2343              new_name = nameMap[self.name] 
2344              if self.multiDefInfo[0]: 
2345                  # then add non-definition version of name to dictionary, 
2346                  # i.e. add z[i] to dictionary containing z[i,0,4] 
2347                  rootname = self.name.split('[')[0] 
2348                  ix = self.multiDefInfo[2] 
2349                  parentname = new_name.split(NAMESEP)[0] 
2350                  nameMap[rootname+'['+ix+']'] = \ 
2351                                   parentname+NAMESEP+rootname+'['+ix+']' 
2352              self.name = new_name 
2353          self.spec.mapNames(nameMap) 
2354          self.spec._check_self_ref() 
2355          self.freeSymbols = remain(self.spec.freeSymbols, self.name) 
2356          self.usedSymbols = self.spec.usedSymbols

2357   
2358   


2359 -    def compileFuncSpec(self):


2360          raise NotImplementedError("Only call this method on a concrete sub-class")

2361   
2362   


2363 -    def simplify(self):


2364          qtemp = self.spec._eval(0) 
2365          self.spec = qtemp 
2366          self.freeSymbols = remain(self.spec.freeSymbols, self.name) 
2367          self.usedSymbols = self.spec.usedSymbols

2368   
2369   


2370 -    def renderForCode(self):


2371          """Return code-ready version of spec (e.g. uncapitalize built-in 
2372          function names)""" 
2373          quant = copy(self) 
2374          quant.spec = self.spec.renderForCode() #._eval(0, {}) 
2375          quant.freeSymbols = remain(quant.spec.freeSymbols, quant.name) 
2376          quant.usedSymbols = quant.spec.usedSymbols 
2377          return quant

2378   


2379 -    def rename(self, new_name):


2380          self.mapNames({self.name: new_name})

2381   


2382 -    def eval(self, *scopearg, **defs):


2383          """eval does not require all free symbols to be resolved, but any 
2384          signature arguments (for a function) must be resolved.""" 
2385          arglist = defs.keys() 
2386          if hasattr(self, 'signature'): 
2387              if not alltrue([s in arglist for s in self.signature]): 
2388                  raise ValueError("All function signature arguments are " 
2389                                   "necessary for evaluation") 
2390          return self.spec._eval(1, *scopearg, **defs)

2391   
2392   


2393 -    def validate(self):


2394          if not isinstance(self.domain, tuple): 
2395              raise TypeError("Expected 3-tuple for domain of token '" \ 
2396                                  + self.name + "'") 
2397          if len(self.domain) != 3: 
2398              raise TypeError("Expected 3-tuple for domain of token '" \ 
2399                                  + self.name + "'") 
2400          if not isinstance(self.spec, QuantSpec): 
2401              raise TypeError("spec field not of type QuantSpec") 
2402          if self.spec.subjectToken != self.name: 
2403              # only raise error if it's not a multiple quantity definition, 
2404              # for which these aren't going to match 
2405              if not isMultiDef(self.name): 
2406                  raise ValueError("spec target mismatch for token '" \ 
2407                               + self.name + "' vs. '" + self.spec.subjectToken \ 
2408                               +"'") 
2409          assert isinstance(self.spec.specStr, str), "specStr invalid" 
2410          assert self.spec.specType in specTypes, "specType invalid" 
2411          bad_tls = remain(self.targetLangs, targetLangs) 
2412          if len(bad_tls)>0: 
2413              print "Invalid target languages:", bad_tls 
2414              raise ValueError("Invalid target language specified") 
2415          if self.typestr != 'var' and self.specType != 'ExpFuncSpec': 
2416              raise TypeError("Invalid spec type specified for %s"%className(self))

2417          # !! check that compatibleGens point to known Generator types !! 
2418          # if get this far without raising exception, then valid 
2419   
2420   


2421 -    def isDefined(self, verbose=False):


2422          self.validate() 
2423          if not self.spec.isDefined(verbose) and self.typestr != 'par': 
2424              if verbose: 
2425                  print "'%s' ill defined: bound spec string is required"%self.name 
2426              return False 
2427          if len(self.compatibleGens) == 0: 
2428              if verbose: 
2429                  print "'%s' ill defined: compatibleGens is empty"%self.name 
2430              return False 
2431          return True

2432   
2433  # bindSpec is broken until it knows how to deal with multi-quantity defs/refs 
2434  #    def bindSpec(self, spec): 
2435  #        if isinstance(spec, QuantSpec): 
2436  #            if spec.subjectToken != self.name: 
2437  #                raise ValueError(self.typestr + " name '" + self.name + "'" \ 
2438  #                             + " does not match '" + spec.subjectToken + "'") 
2439  #            self.spec = spec 
2440  #        else: 
2441  #            raise TypeError("Invalid spec argument") 
2442  #        self.freeSymbols = self.findFreeSymbols()?? 
2443  #        self.specType = spec.specType 
2444  #        self.validate() 
2445   


2446 -    def __repr__(self):


2447          return "%s %s (%s)"%(className(self),self.name,self.specType)

2448   


2449 -    def __str__(self):


2450          return self.name

2451   


2452 -    def info(self, verboselevel=1):


2453          if verboselevel > 0: 
2454              # info is defined in utils.py 
2455              utils_info(self.__dict__, "ModelSpec " + self.name, 
2456                   recurseDepthLimit=1+verboselevel) 
2457          else: 
2458              print self.__repr__()

2459   


2460 -    def __hash__(self):


2461          h = (hash(type(self)), hash(self.name), hash(str(self.spec)), 
2462                 hash(str(self.domain[0])), hash(str(self.domain[1])), 
2463                 hash(str(self.domain[2]))) 
2464          return sum(h)

2465   


2466 -    def __eq__(self, other, diff=False):


2467          results = [] 
2468          try: 
2469              results.append(type(self) == type(other)) 
2470              results.append(self.name == other.name) 
2471              results.extend([str(self.domain[i]) == str(other.domain[i]) \ 
2472                             for i in [0,1,2]]) 
2473              results.append(self.spec == other.spec) 
2474          except AttributeError, e: 
2475              if diff: 
2476                  print "Type:", className(self), results 
2477                  print "  " + e 
2478              return False 
2479          if diff: 
2480              print "Type:", className(self), results 
2481          return alltrue(results)

2482   


2483 -    def __ne__(self, other):


2484          return not self.__eq__(other)

2485   


2486 -    def difference(self, other):


2487          """Show differences in comparison to another Quantity.""" 
2488          self.__eq__(other, diff=True)

2489   
2490  #    def compileFuncSpec(self): 
2491  #        self.funcSpecDict = {self.typestr+'s': [deepcopy(self)]} 
2492   


2493 -    def __copy__(self):


2494          pickledself = pickle.dumps(self) 
2495          return pickle.loads(pickledself)

2496   


2497 -    def __deepcopy__(self, memo=None, _nil=[]):


2498          pickledself = pickle.dumps(self) 
2499          return pickle.loads(pickledself)

2500   
2501  # ------------------------------------------------------------------------ 
2502   


2503 -class Par(Quantity):


2504      typestr = 'par'

2505   
2506   


2507 -class Var(Quantity):


2508      typestr = 'var'

2509   
2510   


2511 -class Input(Quantity):


2512      typestr = 'input'

2513   
2514   


2515 -class Fun(Quantity):


2516      typestr = 'auxfn' 
2517   


2518 -    def __init__(self, spec, signature, name="", domain=None, specType=None):


2519          if not isinstance(signature, _seq_types): 
2520              raise TypeError("signature must be a list/tuple of strings") 
2521          sigstrs = []  # allows signature to be made up of Var names, etc. 
2522          for s in signature: 
2523              # signature may be empty, for function call fn() 
2524              sigstrs.append(str(s)) 
2525              assert isNameToken(str(s)), "signature symbol %s is invalid"%s 
2526          self.signature = sigstrs 
2527          Quantity.__init__(self, spec, name, domain, specType) 
2528          self.freeSymbols = remain(self.freeSymbols, self.signature) 
2529  ##        if remain(self.signature, self.spec.parser.tokenized) != []: 
2530  ##            raise ValueError("All signature arguments were not used in function definition") 
2531          if self.spec.isDefined(): 
2532              protected_auxnamesDB.addAuxFn(self.name, self.spec.parser)

2533   
2534  #    def bindSpec(self, spec): 
2535  #        Quantity.bindSpec(self, spec) 
2536  #        protected_auxnamesDB.addAuxFn(self.name, self.spec.parser, 
2537  #                                      overwrite=True) 
2538   


2539 -    def __call__(self, *args):


2540          lensig = len(self.signature) 
2541          if lensig != len(args): 
2542              raise ValueError("Invalid number of arguments for auxiliary function") 
2543          argstr = ", ".join(map(str,args)) 
2544          if self.spec.specStr == '': 
2545              return QuantSpec("__result__", self.name+'('+argstr+')', specType=self.specType) 
2546          else: 
2547              # avoid name clashes for free names and bound arg names during substitution 
2548              argNameMapInv = {} 
2549              args = [copy(a) for a in args] 
2550              for i in range(len(args)): 
2551                  if isinstance(args[i], (Quantity, QuantSpec)): 
2552                      if args[i].freeSymbols == []: 
2553                          try: 
2554                              check_names = [args[i].name] 
2555                          except AttributeError: 
2556                              check_names = [args[i].subjectToken] 
2557                      else: 
2558                          check_names = args[i].freeSymbols 
2559                      #check_names = args[i].freeSymbols 
2560                      isection = intersect(check_names, self.signature) 
2561                      for name in isection: 
2562                          # dummy arg name 
2563                          newname = "__"+name+"__" 
2564                          while newname in argNameMapInv.keys() + \ 
2565                                  self.signature+self.freeSymbols: 
2566                              # ensure unique name 
2567                              newname += "_" 
2568                          argNameMapInv[newname] = name 
2569                          args[i].mapNames({name:newname}) 
2570                  elif isinstance(args[i], str): 
2571                      qtemp = QuantSpec("__arg__", args[i]) 
2572                      isection = intersect(qtemp.freeSymbols, self.signature) 
2573                      for name in isection: 
2574                          # dummy arg name 
2575                          newname = "__"+name+"__" 
2576                          while newname in argNameMapInv.keys() + \ 
2577                                  self.signature+self.freeSymbols: 
2578                              # ensure unique name 
2579                              newname += "_" 
2580                          argNameMapInv[newname] = name 
2581                          qtemp.mapNames({name:newname}) 
2582                      args[i] = str(qtemp) 
2583              res = self.spec._eval(1, **dict(zip(self.signature,args))) 
2584              res.mapNames(argNameMapInv) 
2585              return res

2586   
2587   
2588  # ------------------------------------------------------------------------- 
2589  ### Finish up overrides for inbuilt math names -> TitleCase symbolic versions 
2590   
2591  # assign math constants to QuantSpecs that hold their names 
2592  for c in remain(allmathnames,funcnames): 
2593      exec(str(c).title() + " = QuantSpec('"+str(c)+"', '', includeProtected=False)") 
2594      math_globals[str(c).title()] = eval(str(c).title()) 
2595   
2596  # references to builtin aux functions or macros require temp defs of those 
2597  # as Fun objects 
2598  local_fndef = {} 
2599  for fname in builtinFnSigInfo.keys(): 
2600      # function will not have to return anything in order to be eval'd 
2601      # but need a return value. we use '0'. 
2602      # final underscore is to avoid name clash with actual Python names 
2603      # when the strings are "eval"'d locally, e.g. "if", "for". 
2604      argstr = "" 
2605      # add signature 
2606      for i in range(builtinFnSigInfo[fname]): 
2607          argstr += "'arg"+str(i)+"'," 
2608      funcstr = "Fun('', [%s], '%s')"%(argstr,fname+"_") 
2609      local_fndef[fname+"_"] = eval(funcstr) 
2610   
2611   
2612  # -------------------------------------------------------------------------- 
2613  ### Symbolic differentiation 
2614   
2615  ##__Diff_saved = {} 
2616  ## 
2617  ##def loadDiffs(filename): 
2618  ##    global __Diff_saved 
2619  ##    try: 
2620  ##        __Diff_saved = cPickle.load(file(filename, 'rb')) 
2621  ##    except IOError: 
2622  ##        pass 
2623  ## 
2624  ##def saveDiffs(filename): 
2625  ##    f = file(filename, 'wb') 
2626  ##    cPickle.dump(__Diff_saved, f, 2) 
2627  ##    f.close() 
2628   
2629   


2630 -def getlftrtD(t,a):


2631      lft=t[1] 
2632      rt=t[3:] 
2633      if len(rt)>1: 
2634          rt=[t[0]]+rt 
2635          drt=DiffStr(rt,a) 
2636          if drt[0] not in ['NUMBER']: 
2637              drt=['atom',['LPAR','('],drt,['RPAR',')']] 
2638      else: 
2639          rt=rt[0] 
2640          drt=DiffStr(rt,a) 
2641      dlft=DiffStr(lft,a) 
2642      return lft,dlft,rt,drt

2643   
2644   


2645 -def dofun(l,r):


2646      retr=ensurebare(r) 
2647      if l=='log': 
2648          if r in ONES: return '0' 
2649          if r=='e': return '1' 
2650      if l=='log10': 
2651          if r in ONES: return '0' 
2652          if r in TENS: return '1' 
2653      if l=='log_0': return dodiv('1',r) 
2654      if l=='log10_0': return dodiv(dofun('log','10'),retr) 
2655      if l=='ln_0': return dodiv('1',r) 
2656      if l=='sin_0': return dofun('cos',retr) 
2657      if l=='sinh_0': return dofun('cosh',retr) 
2658      if l=='cosh_0': return dofun('sinh',retr) 
2659      if l=='tanh_0': return dodiv('1',dopower(dofun('cosh',retr),'2')) 
2660      if l=='coth_0': return dodiv('-1',dopower(dofun('sinh',retr),'2')) 
2661      if l=='asin_0': return dodiv('1',dofun('sqrt',dosub('1',dopower(r,'2')))) 
2662      if l=='acos_0': return dodiv('-1',dofun('sqrt',dosub('1',dopower(r,'2')))) 
2663      if l=='atan_0': return dodiv('1',doadd('1',dopower(r,'2'))) 
2664      if l=='acot_0': return dodiv('-1',doadd('1',dopower(r,'2'))) 
2665      if l=='tan_0': return dodiv('1',dopower(dofun('cos',retr),'2')) 
2666      if l=='cot_0': return doneg(dodiv('1',dopower(dofun('sin',retr),'2'))) 
2667      if l=='cos_0': return doneg(dofun('sin',retr)) 
2668      if l=='exp_0': return dofun('exp',retr) 
2669      if l=='sqrt_0': return dodiv('1',domul('2',dofun('sqrt',retr))) 
2670      return '%s(%s)'%(l,retr)

2671   
2672   
2673   
2674   
2675  # -------------------------------------------------------------------------- 
2676   
2677  qtypes = (Quantity, QuantSpec) 
2678   


2679 -def Diff(t, a):


2680      """Diff expects strings, Quantity, or QuantSpec types, 
2681      a mixture of these, or a list of strings in second argument. 
2682   
2683      The return type is a QuantSpec. 
2684      """ 
2685   
2686      # deal with t ----------------------------------------- 
2687      if compareClassAndBases(t, qtypes): 
2688          if t.isvector(): 
2689              return Diff(t.fromvector(), a) 
2690          qt = copy(t) 
2691          qt.mapNames(feval_map_symb) 
2692          if isinstance(t, Fun): 
2693              # shorthand so that function f can be diff'd without having to 
2694              # specify f(x,y,z) (i.e. without signature) 
2695              t_arg = str(qt(*([str(qarg) for qarg in qt.signature]))) 
2696          else: 
2697              try: 
2698                  # extract symbol definition, if any 
2699                  t_arg = qt.spec.specStr 
2700              except AttributeError: 
2701                  t_arg = str(qt) 
2702              else: 
2703                  if t_arg == '': 
2704                      # use symbol name 
2705                      t_arg = str(qt) 
2706      elif isinstance(t, str): 
2707          t_strip = t.strip() 
2708          if t_strip[0] == "[" and t_strip[-1] == "]": 
2709              return Diff(splitargs(t_strip[1:-1]), a) 
2710          else: 
2711              qt = QuantSpec('qt', t, treatMultiRefs=False) 
2712              qt.mapNames(feval_map_symb) 
2713              t_arg = str(qt) 
2714      elif compareClassAndBases(t, (list, tuple)): 
2715          ostr = "[" + ",".join([str(Diff(o,a)) for o in t]) + "]" 
2716          return QuantSpec("__result__", ostr) 
2717      else: 
2718          print "Found:", t, "type (%s)"%type(t) 
2719          raise TypeError("Invalid type for t") 
2720      # deal with a ----------------------------------------- 
2721      if compareClassAndBases(a, qtypes): 
2722          a_arg = str(a) 
2723      elif isinstance(a, list): 
2724          ostr = "[" + ",".join([str(Diff(t,o)) for o in a]) + "]" 
2725          return QuantSpec("__result__", ostr) 
2726      elif isinstance(a, str): 
2727          a_arg = a 
2728      else: 
2729          print "Found:", a, "type (%s)"%type(a) 
2730          raise TypeError("Invalid type for a") 
2731      # if didn't already recurse then fully reduced to strings 
2732      # ... attempt to simplify string using eval() method call 
2733      try: 
2734          res = QuantSpec("__result__", DiffStr(t_arg, a_arg)) 
2735          res.mapNames(inverseMathNameMap) 
2736          res.simplify() 
2737          return res 
2738      except: 
2739          print "Tried to diff '%s' w.r.t. '%s'"%(t_arg,a_arg) 
2740          raise

2741   
2742   


2743 -def DiffStr(t, a='x'):


2744      """Diff for strings only. 
2745   
2746      This is Pearu's original Diff function renamed to DiffStr.""" 
2747   
2748      if isinstance(a, list): 
2749  ##        s= ''.join([t, '__derivWRT__', str(a)]) 
2750  ##        r=__Diff_saved.get(s, None) 
2751  ##        if r is not None: 
2752  ##            return r 
2753   
2754          o = string2ast(t) 
2755          for aa in a: 
2756              o = DiffStr(o,aa) 
2757  ##        res=ast2string(o) 
2758  ##        __Diff_saved[s] = res 
2759  ##        return res 
2760          return ast2string(o) 
2761      elif isinstance(t, str): 
2762          return ast2string(DiffStr(string2ast(t),a)) 
2763  ##        s= ''.join([t, '__derivWRT__', str(a)]) 
2764  ##        r=__Diff_saved.get(s, None) 
2765  ##        if r is not None: 
2766  ##            return r 
2767  ## 
2768  ##        res=ast2string(DiffStr(string2ast(t),a)) 
2769  ##        __Diff_saved[s] = res 
2770  ##        return res 
2771   
2772      if isinstance(t, list) and len(t) == 1: 
2773          return DiffStr(t[0],a) 
2774      if t[0]=='NUMBER': return [t[0],'0'] 
2775      if t[0]=='NAME': 
2776          if t[1]==a: return ['NUMBER','1'] 
2777          return ['NUMBER','0'] 
2778      if t[0]=='factor': 
2779          st=ast2string(t) 
2780          return simplify(string2ast(doneg(ast2string(DiffStr(string2ast(st[1:]),a))))) 
2781      if t[0]=='arith_expr': 
2782          o='0' 
2783          for i in range(1,len(t),2): 
2784              if t[i-1][1]=='-': 
2785                  o=doadd(o,doneg(ast2string(DiffStr(t[i],a)))) 
2786              else: 
2787                  o=doadd(o,ast2string(DiffStr(t[i],a))) 
2788          return simplify(string2ast(o)) 
2789      if t[0]=='term': 
2790          ttemp = ensureparen_div(t) 
2791          lft,dlft,rt,drt=map(lambda x: ast2string(simplify(x)), 
2792                              getlftrtD(ttemp,a)) 
2793          if ttemp[2][0]=='STAR': 
2794              return simplify(string2ast(doadd(domul(dlft,rt),domul(lft,drt)))) 
2795          if ttemp[2][0]=='SLASH': 
2796              return simplify(string2ast(dosub(dodiv(dlft,rt), 
2797                                      domul(domul(lft,drt),dopower(rt,'-2'))))) 
2798      if t[0]=='xor_expr' and t[2]=='CIRCUMFLEX': # covers alternative power syntax 
2799          alt = copy(t) 
2800          alt[0] = 'power'; alt[2]=='DOUBLESTAR' 
2801          return simplify(toCircumflexSyntax(string2ast(DiffStr(alt,a)))) 
2802      if t[0]=='power': 
2803          # covers math functions like sin, cos, and log10 as well! 
2804          if t[2][0]=='trailer': 
2805              if len(t)>3: 
2806                  term1 = simplify(t[:3]) 
2807                  if term1[0] in ['power', 'NUMBER', 'NAME']: 
2808                      formatstr='%s' 
2809                  else: 
2810                      formatstr='(%s)' 
2811                  return simplify(string2ast(DiffStr([t[0], 
2812                                      string2ast(formatstr%ast2string(term1)),t[3:]],a))) 
2813              elif len(t)==3 and t[1][1]=='pow': 
2814                  # 'pow' syntax case 
2815                  return simplify(toPowSyntax(string2ast(DiffStr(mapPowStr(t),a)))) 
2816              # else ignore and carry on 
2817          ts=[];o=[];dts=[] 
2818          for i in t[1:]: 
2819              if i[0]=='DOUBLESTAR': 
2820                  if len(o)==1: o=o[0] 
2821                  ts.append(o); 
2822                  dts.append(DiffStr(o,a)) 
2823                  o=[] 
2824              else: o.append(i) 
2825          if len(o)==1: o=o[0] 
2826          ts.append(o) 
2827          dts.append(DiffStr(o,a)) 
2828          if t[2][0]=='DOUBLESTAR': 
2829              st,lft,dlft,rt,drt=map(lambda x: ast2string(simplify(x)), 
2830                                     [t,ts[0],dts[0],ts[1],dts[1]]) 
2831              rt1=trysimple('%s-1'%rt) 
2832              if drt=='0': 
2833                  return toPowSyntax(string2ast(domul(domul(rt,dlft),dopower(lft,rt1)))) 
2834              return toPowSyntax(string2ast(domul(doadd(domul(dofun('log',lft),drt), 
2835                                            dodiv(domul(dlft,rt),lft)),st))) 
2836          if t[2][0]=='trailer': 
2837              return simplify(toPowSyntax(dts[0])) 
2838      if t[0] in ['arglist','testlist']: 
2839          o=[] 
2840          for i in t[1::2]: 
2841              o.append(DiffStr(ast2string(i),a)) 
2842          return string2ast(','.join(o)) 
2843      if t[0]=='atom': 
2844  ##        if t[1][0]=='LPAR' and t[-1][0]=='RPAR': 
2845  ##            return simplify(DiffStr(t[2:-1],a)) 
2846  ##        else: 
2847          return string2ast(ensureparen(ast2string(DiffStr(t[2:-1],a)))) 
2848      if t[1][0]=='trailer': # t=[[NAME,f],[trailer,[(],[ll],[)]]] 
2849          aa=t[1][2] 
2850          ll=splitargs(ast2string(DiffStr(aa,a))) 
2851          ii=0;o='0' 
2852          for i in ll: 
2853              o=doadd(o,domul(i,dofun(t[0][1]+'_'+`ii`,ast2string(aa)))) 
2854              ii=ii+1 
2855          return simplify(string2ast(o)) 
2856      return t

2857
```

  


| Home | Trees | Indices | Help | PyDSTool |
| --- | --- | --- | --- | --- |

|  |  |
| --- | --- |
| Generated by Epydoc 3.0.1 on Fri May 4 15:24:16 2012 | http://epydoc.sourceforge.net |
